# Predicting Emergency Severity Index (ESI) level, hospital admission, and admitting ward in an emergency department using data-driven machine learning

**DOI:** 10.1186/s12911-025-02941-9

**Published:** 2025-07-28

**Authors:** Steve Agius, Vincent Cassar, Caroline Magri, Wasiq Khan, Dhiya Al-Jumeily Obe, Godwin Caruana, Luke Topham

**Affiliations:** 1 University of Malta, Msida, Malta; 2 Liverpool John Moores University, Liverpool, UK

**Keywords:** Emergency department, Triage, Big-data, Machine learning, Decision-making, XGBoost

## Abstract

**Introduction:**

Emergency departments (EDs) are critical for ensuring timely patient care, especially in triage, where accurate prioritisation is essential for patient safety and resource utilisation. Building on previous research, this study leverages a comprehensive dataset of 653,546 ED visits spanning six years from Mater Dei Hospital, Malta. This dataset enables detailed trend analysis, demographic variation exploration, and predictive modelling of patient prioritisation, admission likelihood, and admitting ward.

**Methods:**

Two predictive models (Stage 1 and Stage 2) were developed using the Extreme Gradient Boosting (XGBoost) algorithm. In Stage 1, predictions were made at the triage level using basic demographic and presenting symptom data. Stage 2 incorporated critical blood test results (e.g., Haemoglobin, C-Reactive Protein, Troponin T, and White Blood Cell Count) alongside the demographic and symptom data from Stage 1 to refine and enhance predictions.

Key steps in data preprocessing, such as handling missing values, balancing class distributions with SMOTE, and feature encoding, are discussed. Model evaluation employed comprehensive metrics, including AUC-ROC and calibration curves, to assess both performance and reliability. This enhanced description provides a clear roadmap of the model development process, reinforcing the study’s rigor and contribution to advancing machine learning applications in emergency care.

**Results:**

The models demonstrated significant predictive capabilities. Key metrics showed improvement between Stage 1 and Stage 2. For example, patient prioritisation accuracy improved from 0.75 to 0.76, admission prediction accuracy rose from 0.80 to 0.82, and admitting ward prediction accuracy increased from 0.80 to 0.86. These enhancements underscore the value of incorporating clinical data to optimise predictions.

**Discussion:**

The integration of early predictions into ED workflows has the potential to improve patient flow, reduce wait times, and enhance resource allocation. By leveraging XGBoost’s capabilities and integrating both demographic and clinical data, this study provides a robust framework for advancing decision-making processes in triage environments.

**Conclusions:**

This research demonstrates the efficacy of machine learning models in predicting key ED outcomes, highlighting their potential to transform emergency care through data-driven insights.

**Supplementary Information:**

The online version contains supplementary material available at 10.1186/s12911-025-02941-9.

## Background

The emergency department (ED) is one of the most important components of a hospital ecosystem [1] and plays a vital role in saving people’s lives and reducing the rate of mortality and morbidity [2]. It is a critical interface between the emergency medical services and the hospital [3]. EDs have no patients of their own but serve as a portal of entry to other specialised departments and wards within the hospital [4].

EDs are among the highest-risk areas within any hospital, where the emergency team faces constant challenges such as high workloads, simultaneous care of multiple patients, and frequent overcrowding [5, 6]. Patients presenting themselves at the ED are usually in critical condition and require immediate attention [7]. Healthcare professionals working in the ED are subject to several operation constraints and have to assemble and manage unrehearsed multidisciplinary teams with little notice and manage critically ill patients [5]. Patients are assessed, classified, and prioritised according to their medical condition in a restricted time-window [8]. This process of classification and prioritisation, known as triage, primarily aims to organise the work of the ED for greater efficiency and optimal resource utilisation. More importantly, it promotes patient safety by ensuring that care and resource allocation are aligned with the level of severity of illnesses [3, 9]. This creates an environment that is not only highly complex and dynamic, but also functions under extreme constraints of time, physical space, high workload, interruptions, and distractions, with a significant level of uncertainty [10].

Improper triaging and prioritisation of patients can result in delayed care due to postponements or deferrals in giving treatment and inappropriate assignment of resources [11]. In ED, where the majority of patients are unknown, and their illnesses are seen through only small windows of focus and time [8], triage nurses are situated in high levels of uncertainty, which poses serious risks associated with inaccurate or inappropriate decisions [12]. These decisions affect patients’ well-being, and while most of the time these decisions are correct, sometimes they can be inappropriate, leading to fatal results [13]. As a result, the ED has been identified as a hospital location where adverse events are highly likely to be attributable to errors [14]. Estimates of the proportion of ED adverse events deemed to be preventable range from 53 to 82% compared with overall estimates of 27% to 51% for hospital-based adverse events [15–17].

Clinical decision support systems can assist triage nurses in decision-making by providing patient-specific assessments or recommendations [18–21]. These systems are designed to improve both the process and the outcome of medical decision-making [22] with targeted clinical knowledge, patient information, and other health information [23]. Their purpose is to augment the natural capabilities of the triage nurse in the complex process of medical diagnosis by improving triage accuracy [24], increased efficiency [25], reduce wait times [26], enhance patient safety [27] and improve resource allocation [28].

Previous studies have sought to predict ESI level [29] and hospital admission [30, 31] at the time of ED triage using machine learning models. Most models use routine administrative data collected at emergency triage and can robustly predict both ESI level and hospital admission. The addition of historical information such as lab test results, medications prescribed, and comorbidities has been able to achieve high predictive power and indicates the utility of these additional data points [32, 33].

This study aims to fill this gap by leveraging a novel, comprehensive dataset encompassing all ED visits across Malta from 2007 to 2022. The primary objective is to develop machine learning models that predict patient prioritisation, hospital admission likelihood, and admitting ward categorisation, thereby improving triage accuracy and resource allocation. By integrating both demographic and clinical data, the study seeks to provide actionable insights to optimise emergency care delivery on a national scale.

### Study overview

Building on the methodologies of previous research [30, 32, 34–37] this study makes a novel contribution through its findings and extensive scope and methodology. Unlike earlier studies limited to specific hospitals or regions, this research utilises a comprehensive dataset covering all ED visits across Malta from 2007 to 2022. The methodological rigor of this study is significantly shaped by insights from a preceding study that combined intuitive and analytical decision-making in emergency triage, which helped address critical challenges such as time constraints and diagnostic uncertainty. This previous study’s influence is evident in the enhanced decision-making framework applied here, ensuring more precise and reliable predictions in emergency triage settings.

### Study scope

The study utilises data exclusively from Mater Dei Hospital, Malta, the primary public hospital on the island, providing a comprehensive national dataset encompassing all ED visits across the country. This geographic scope ensures that the data reflects healthcare utilisation patterns across the entire Maltese population. Temporally, the dataset spans six years, from January 2017 to December 2022, capturing over 653,000 ED visits. This extensive timeframe allows for the identification of longitudinal trends and patterns in patient care, resource utilisation, and changes in healthcare delivery, including shifts induced by external factors such as the COVID-19 pandemic.

This comprehensive national dataset not only enhances the statistical power of the findings but also significantly improves their generalisability to diverse populations. By encompassing a complete overview, the study allows for more accurate identification of trends in healthcare utilisation and patient outcomes across Malta, offering insights that are reflective of the real-world complexities of healthcare system interactions and patient care patterns.

By including all ED visits nationwide, the research enables detailed comparisons with international studies and provides a solid basis for understanding national healthcare trends. Observational findings, such as those from the Southern Harbour region which exhibited high rates of ED visits for chest pain potentially linked to economic factors [38] underscore the dataset’s value in exploring demographic variations and informing healthcare policy. The longitudinal nature of the data also facilitates studies of shifts in healthcare practices and patient behaviour, including those induced by the COVID-19 pandemic.

Leveraging such a rich dataset enables invaluable insights into ED utilisation patterns, healthcare delivery, and patient outcomes on a national scale. The comprehensive nature of this research makes it a valuable addition to both academic research and practical healthcare strategy development, providing critical insights into disparities and identifying areas for targeted interventions.

## Methodology

**Fig. 1:**
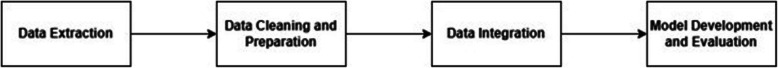
Workflow of this research study

This section describes the study’s methodology, designed to ensure reproducibility, transparency, and clarity. Figure 1 provides a structured outline of the steps taken from data extraction to model development and evaluation.

### Data extraction

Data were retrospectively collected from the Health Information System (HIS) of Mater Dei Hospital, encompassing all ED visits across Malta from 2007 to 2022. This foundational step involved gathering extensive demographic information such as gender, age, and geographic region, as well as clinical data, including results from blood tests taken at the ED. Such comprehensive data collection is critical for building a dataset that accurately reflects the true scope of healthcare dynamics and patient interactions within the ED. With 32,373,603 individual data points, this expansive dataset not only offers numerous advantages for investigating healthcare outcomes but also significantly enhances statistical power. This robustness in the data increases the reliability and confidence in the results obtained from subsequent analyses.

### Data cleaning and preparation

Data cleaning was the initial step in the process, involving the standardisation of data formats and the removal of extraneous characters to enhance data quality, a fundamental requirement for accurate analysis and integration. During the data integration phase, diverse data elements from multiple sources within the hospital’s HIS were linked using unique patient identifiers, ensuring the maintenance of data consistency and integrity across the dataset. To safeguard privacy in compliance with GDPR and ensure the integrity of data integration, sensitive patient data underwent pseudo-anonymisation through tokenization. Additionally, the dataset was further refined during the feature engineering phase by addressing missing values and ensuring uniformity in data entries, which are crucial for the reliability of machine learning models. This phase involved the creation of new features and the modification of existing ones to better capture the underlying patterns needed for analysis.

### Data integration

After cleaning, the data from various sources were integrated into a unified dataset using a unique patient identifier (PID). This integration ensures accurate data consolidation while maintaining patient confidentiality, allowing for a holistic view of each patient’s journey through the ED. The consolidated data then formed a unified data source, serving as the backbone for all further analyses and model training. This dataset contains all necessary variables and historical data points required for robust predictive modelling, providing a comprehensive dataset for subsequent analysis.

### Model development and evaluation

The study involved the development and optimisation of four predictive models using two distinct sets of variables: basic models utilising only demographic and administrative data, and enhanced models incorporating additional clinical and laboratory variables. The algorithm used was XGBoost, selected for its efficacy in managing imbalanced datasets [39]. To address class imbalances, the Synthetic Minority Over-sampling Technique (SMOTE) was applied to enhance model robustness. SMOTE helps balance the dataset by synthetically generating new instances of the minority class, improving model generalisation and accuracy by ensuring equitable class representation in the training data. This adjustment prevents model bias towards the majority class, making the predictions more reliable across diverse scenarios. The performance of these models was rigorously evaluated using a variety of metrics including accuracy, precision, recall, F1-scores, AUC-ROC curves, and calibration curves to ensure prediction reliability.

In terms of predictive modelling, these models were carefully calibrated to meet the specific needs of emergency care settings, predicting patient prioritisation, hospital admission, and specialty ward assignments. Extensive validation and testing were conducted post-development to ensure accuracy and generalisability, confirming the models’ reliability across various patient scenarios and their suitability for clinical application.

The computational framework for these analyses relied heavily on Python, using libraries such as Pandas for data manipulation and cleaning, which is crucial for managing large datasets; NumPy for supporting complex numerical calculations; SciKit-Learn for implementing and evaluating machine learning algorithms and which aided in model tuning and validation; and XGBoost for its gradient boosting capabilities, which significantly enhance model performance and prevent overfitting. This comprehensive suite of tools ensured that the statistical analyses and model training were conducted efficiently and effectively.

## Data processing and integration

Data was extracted from diverse sources to ensure a comprehensive and representative dataset. Given the heterogeneity of the data sources, a comprehensive data cleansing process was carried out to enhance data quality and retain data integrity. This process included the removal of extraneous characters, normalisation of data formats, and enforcing uniform data standards to ensure consistency across the dataset. Additionally, several records were further standardised to establish a more structured and uniform dataset. Once the data was thoroughly cleaned and consolidated into a unified data source, pseudo-anonymisation was applied using tokenisation techniques to protect patient identities.

Tokenisation was selected for this dataset because it replaces sensitive patient information with non-sensitive equivalents (tokens), ensuring privacy while maintaining the data’s usability for analysis [40]. This approach ensures that patients were not recognisable, as tokenisation took place prior to data analysis, further safeguarding their privacy. Tokenisation offers a robust method for de-identifying data in compliance with GDPR regulation, without compromising the integrity of clinical data required for research and analysis [41].

Following the data tokenisation process, the data underwent rigorous testing for consistency and quality assurance, including data validation to ensure tokenised values maintained referential integrity across the dataset, as well as cross-validation to confirm that the tokenisation did not inadvertently alter or obscure critical data attributes. Additionally, both functional and non-functional testing were conducted to assess the security and performance of the tokenised data, ensuring compliance with healthcare privacy standards and the continued accuracy of the dataset for analytical purposes. Finally, the prepared dataset was exported in Comma Separated Variable (CSV) format and securely stored in an encrypted repository to ensure data protection and compliance with regulatory standards.

In total, the dataset comprises 653,546 ED visits, equivalent to 257,495 unique patients. Each record contains 21 variables that capture clinical, demographic, and healthcare-related information. The variables used in this study are explained in detail in Appendix 1.

The dataset preparation involved replacing missing values with zeros or 'MISSING' labels to maintain the integrity of the dataset and avoid data loss. This straightforward imputation method allows XGBoost to effectively handle incomplete records, as the algorithm is capable of learning from missing data patterns. By explicitly flagging missing values, the model can differentiate between actual data and missing entries, ensuring that these gaps do not adversely affect predictive accuracy while simplifying the preprocessing stage. The target variables (Patient Prioritisation, Patient Admission, Main Category Admitting Ward and Subcategory Admitting Ward) were then encoded using LabelEncoder, a technique that converts categorical columns into numerical values, enabling them to be used by machine learning models, which only accept numerical data. This preprocessing step is essential in machine learning projects, as models like XGBoost require numerical input.

To enhance the accuracy of the predictive model for the admission ward, a second copy of the dataset was created, including only those patients who had been admitted to the hospital over the same six-year period. This focused approach narrows the scope of the model to a subset of patients where outcomes such as hospital admission are more relevant. By concentrating on admitted patients, the model can better identify critical patterns that influence hospital admissions, which would be diluted if non-admitted patients were included. This refinement ensures that the model is trained on the most pertinent data, improving its ability to accurately predict which patients require admission.

## Model development: XGBoost

The predictive model adopted in this study is based on the Extreme Gradient Boosting (XGBoost) Classifier. XGBoost is a powerful machine learning algorithm that gained recognition for its high performance in predictive modelling, particularly with large, complex, and often imbalanced datasets typical present in healthcare research [42]. As an advanced form of gradient boosting, XGBoost builds a collection of decision trees sequentially, where each tree attempts to correct the errors made by its predecessors [42]. This method employs a regularised objective function that balances model accuracy and complexity, helping to reduce overfitting.

One of XGBoost’s strengths is its incorporation of second-order optimisation, which utilises both the gradient (first derivative) and Hessian (second derivative) of the loss function [39]. This approach allows for more precise and stable updates during training, improving convergence speed and enhancing overall model performance. XGBoost effectively handles both categorical and numerical features using one-hot encoding and offers built-in support for missing values. It uses a sparsity-aware algorithm to manage missing data by learning the optimal imputation strategy that minimises the loss [43]. This feature is particularly beneficial in healthcare datasets, where incomplete records are common [44].

XGBoost is also designed to handle class imbalance [39], a frequent challenge in healthcare datasets where certain outcomes, such as ordering a particular blood test, have fewer instances than others. The algorithm addresses this through parameters like scale_pos_weight, which adjusts the model’s focus on minority classes, and through the integration of techniques like the Synthetic Minority Over-sampling Technique (SMOTE) to further enhance the model’s ability to accurately predict underrepresented outcomes.

Furthermore, XGBoost generates feature importance scores, offering insights into which variables most significantly influence predictions. This feature supports healthcare researchers by highlighting key factors driving prediction outcomes. The algorithm also includes tree pruning based on complexity scores, controlling tree growth and improving model interpretability. Its computational efficiency, scalability, and capacity for managing complex relationships in heterogeneous datasets make XGBoost a robust tool for clinical decision support. It has been successfully applied to predict patient admissions, disease progression, and treatment outcomes, enabling real-time, accurate decision-making in emergency settings. These attributes make XGBoost an asset in healthcare research, striking a critical balance between predictive accuracy and model interpretability.

### Model configuration

**Fig. 2:**
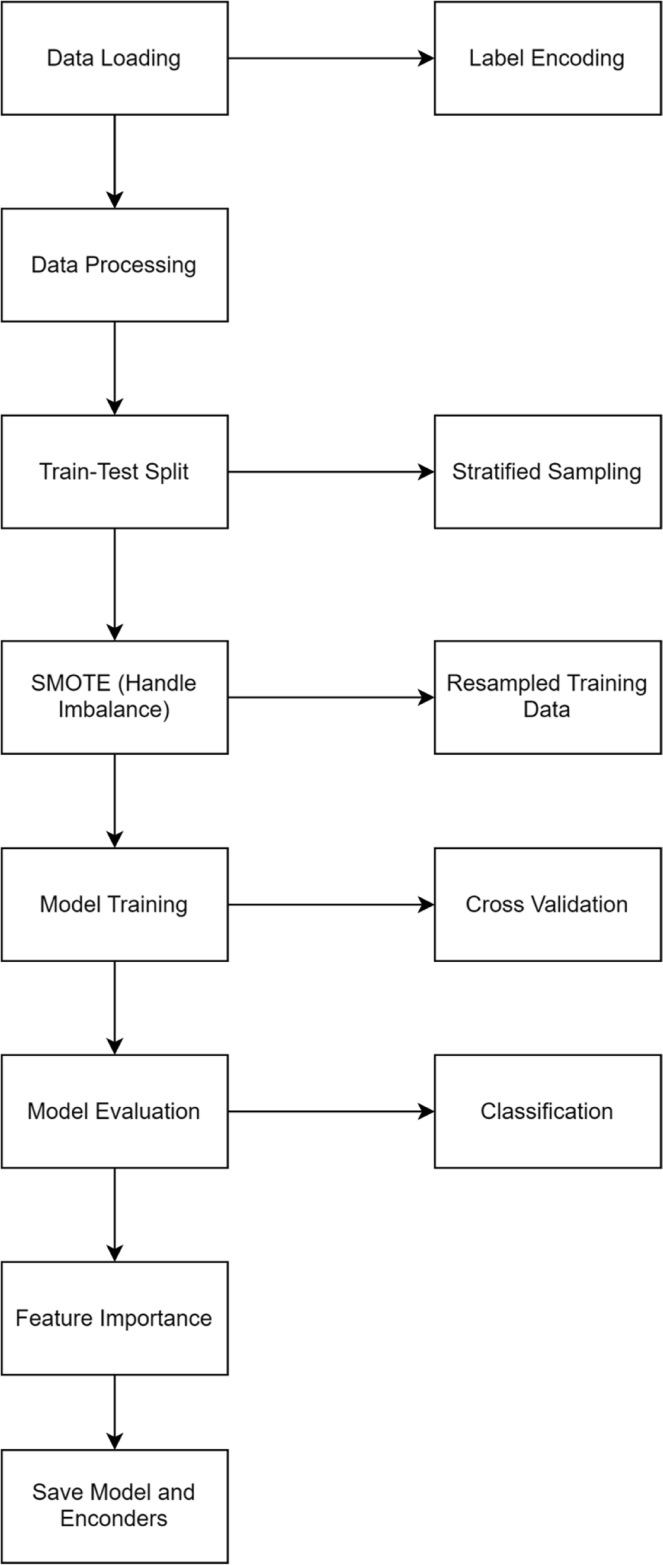
Model architecture

The architecture for the XGBoost-based machine learning method follows a structured process, starting from data preprocessing, handling class imbalance, model training, evaluation, and model saving. Figure 2 visually represents each component from the initial preprocessing stages to model evaluation and deployment.

The first step in implementing the prediction algorithm is the data loading and preprocessing stage, where the dataset is loaded using Pandas,^1^ and relevant columns are filtered for analysis. Missing values are amputated, as explained in the previous sections, ensuring no data is omitted during the training process. The target variable (Patient Prioritisation, Patient Admission, Main Category Admitting Ward and Subcategory Admitting Ward) is label-encoded, and categorical features are one-hot encoded to convert them into a format suitable for machine learning algorithms. These transformations, along with the processed dataset, are saved for future use, enabling consistent application in later predictions.

Once preprocessing is complete, the train-test split is performed, where the dataset is divided into training and test sets. In most cases, a typical ratio for this split is 80:20 or 70:30, where 80% or 70% of the data is used for training, and the remaining 20% or 30% is reserved for testing [45]. However, this ratio may vary depending on the size and characteristics of the dataset. In this study, an 80:20 ratio was utilised for the implementation of XGBoost. This split was chosen to ensure a balance between adequately training the model and evaluating its performance. With 80% of the data used for training, the model has sufficient exposure to learn patterns and relationships within the dataset, which is especially crucial for complex healthcare data. The remaining 20% provides enough data to reliably assess the model’s generalisation ability without risking overfitting.

To address the issue of class imbalance, Synthetic Minority Over-sampling Technique (SMOTE) is applied to the training data. SMOTE generates synthetic samples for the minority class, allowing the model to learn more effectively from underrepresented cases. This technique is particularly beneficial in healthcare applications, where minority classes often represent critical but infrequent conditions. SMOTE ensures that the model is trained with a more balanced dataset, reducing the risk of overfitting and improving its ability to generalise across all classes.

Following data preparation, the model is trained using XGBoost, a high-performance gradient boosting algorithm. Key parameters (outlined in Table 1) are fine-tuned to optimise the model, including setting the learning rate at 0.05, fixing n_estimators at 500, and configuring max_depth at 8 to control model complexity. Additionally, scale_pos_weight is adjusted to 1.65 to handle any residual imbalance after SMOTE. A fivefold stratified cross-validation approach is employed to further enhance the model’s robustness. This method ensures that class proportions are preserved within each fold, providing a reliable estimate of how well the model generalises to unseen data. A fixed random_state of 42 is used throughout the process, ensuring reproducibility of results, which is crucial for validating model performance. This is essential for verifying and comparing the model’s performance, where reproducibility of results is critical. While there is no technical reason for choosing this number over others, it has become popular in the programming and data science community [46].

**Table 1:** XGBoost parameter configuration

Component	Parameter	Value
train_test_split	test_size	0.20
train_test_split	random_state	42
train_test_split	stratify	y_esi
SMOTE	random_state	42
XGBClassifier	random_state	42
XGBClassifier	eval_metric	Mlogloss
XGBClassifier	scale_pos_weight	406596 / 246949 (approx. 1.65)
XGBClassifier	learning_rate	0.05
XGBClassifier	n_estimators	500
XGBClassifier	max_depth	8
XGBClassifier	subsample	0.80
XGBClassifier	colsample_bytree	0.80
StratifiedKFold	n_splits	5
StratifiedKFold	shuffle	TRUE
StratifiedKFold	random_state	42

Model evaluation is conducted using the test data. The model’s performance is assessed using classification metrics such as accuracy, precision, recall, and F1-score, offering insights into its ability to generalise across various classes. Maintaining class balance during evaluation is particularly important in imbalanced datasets to ensure that minority classes are adequately represented in the results, enhancing the model’s reliability.

To gain further insights into the model’s decision-making process, XGBoost’s built-in feature importance metric is employed. This metric ranks features based on their contribution to the model’s predictions, allowing for a detailed understanding of how clinical, demographic, and healthcare-related features influence the model’s outcomes. Such insights are crucial in healthcare applications, where transparency and explainability are vital for trust in predictive models. Clinicians need to not only understand the predictions but also the factors driving them. By identifying and ranking the most important features, the model provides an explanation for its predictions, ensuring that critical clinical or demographic variables are appropriately considered. This level of explainability is key to ensuring fairness, as it helps detect and mitigate potential biases, guaranteeing equitable treatment across all patient groups.

Finally, the trained model, along with the label encoders and encoded feature columns, is saved using joblib, ensuring that the model is reusable for real-time applications in future scenarios. This ensures that the model, once trained, can be seamlessly deployed or further refined without the need for retraining.

The AUC-ROC (Area Under the Receiver Operating Characteristic) curve was utilised in this study as a robust statistical tool to evaluate the diagnostic accuracy and performance of the predictive models. The ROC curve is a graphical representation that plots the true positive rate (sensitivity) against the false positive rate (1-specificity) across various threshold values, illustrating the trade-offs between sensitivity and specificity for each cut-off point. The area under this curve (AUC) quantifies the model’s ability to distinguish between classes, with a value closer to 1 indicating a high level of accuracy. This method is particularly valuable in healthcare especially in medical diagnostics where continuous test results are converted into dichotomous outcomes (e.g., presence or absence of a condition), and finding the optimal threshold is crucial [47]. By providing a comprehensive evaluation of model performance, the AUC-ROC allows for comparison of different models and assists in selecting the most effective diagnostic approach, ensuring a balanced consideration of both sensitivity and specificity.

In addition, calibration curves were added to further assess the reliability of the model’s probability outputs [48]. These curves, which plot the fraction of true outcomes versus the model’s predicted probabilities, help to visually evaluate how well the probabilities estimated by the model correspond to the actual results. For a perfectly calibrated model, the curve would form a 45-degree line indicating that the predicted probabilities are identical to the actual outcomes. This calibration plot is crucial for understanding how well the predicted probabilities correspond to the actual outcomes, highlighting areas where the model’s confidence in its predictions aligns with the observed frequencies [49]. By addressing these discrepancies, the model can be fine-tuned to improve its reliability, which is particularly important in medical settings where precise risk estimation is critical for patient management and treatment decisions.

## Results

This section starts by outlining the process patients undergo upon arrival at the ED. Upon arrival, patients first register at the registration desk, where they provide their personal details and describe their presenting symptoms. This information is entered into the Health Information System (HIS) and is made accessible at the triage nurse stations. The triage nurse then calls the patient to the triage area for an initial clinical assessment, where the urgency of the patient’s condition is evaluated, and a triage level is assigned. During this assessment, the nurse assigns a triage level based on the urgency of the patient’s condition. After triage, the patient moves on to medical evaluation for diagnosis and stabilisation before being either admitted to the hospital or discharged. On average, the entire process from admission to either discharge or admission takes approximately 8 to 10 h [50].

**Fig. 3:**
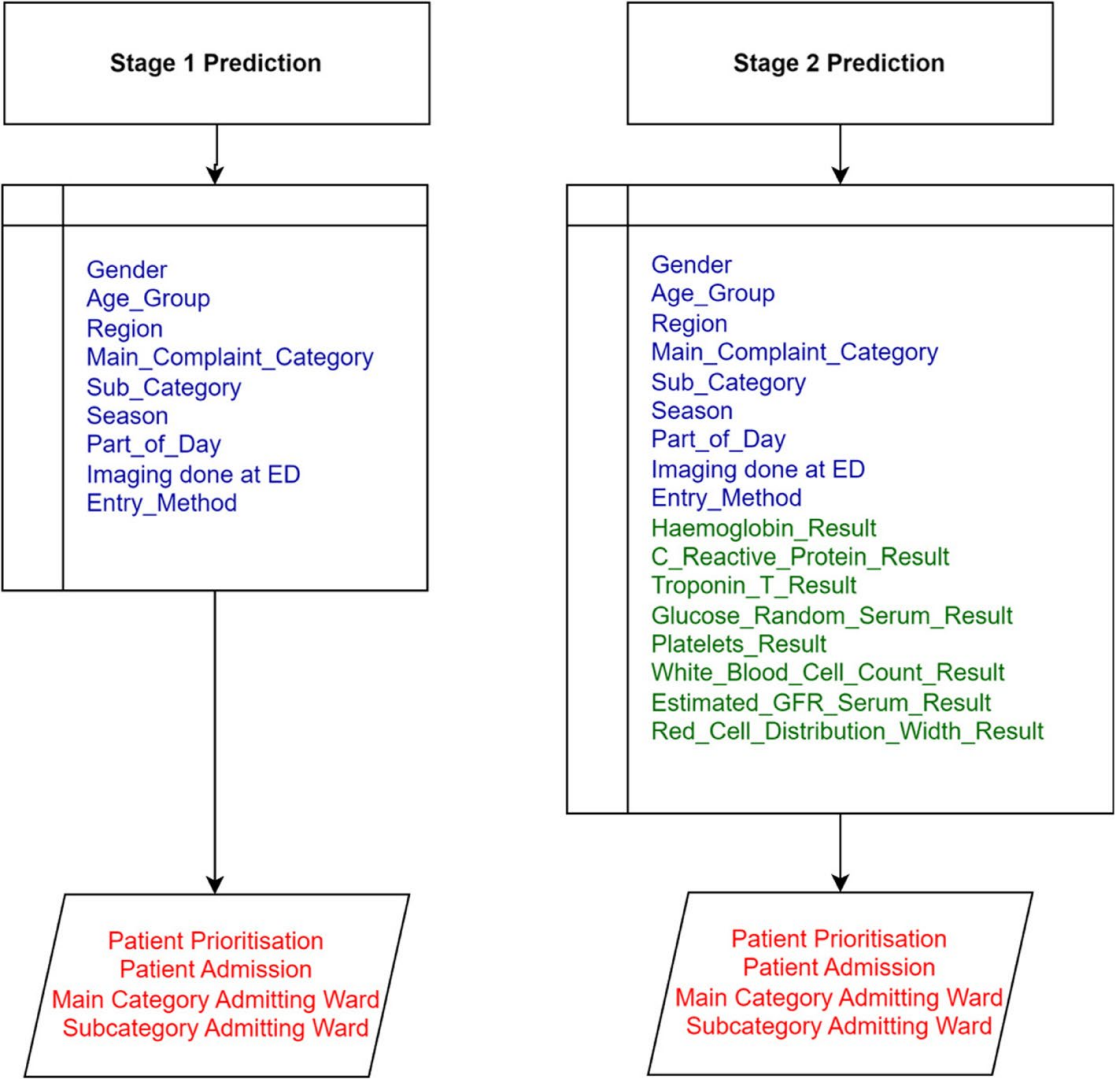
Prediction model (Two staged model)

The prediction model presented in this study and as outlined in Fig. 3 is split into two distinct stages: the first stage that involves generating an initial prediction at the triage level using readily available basic patient data, including demographic information and presenting symptoms. This early prediction provides a preliminary assessment of the patient’s condition. The second stage occurs at a later stage when critical blood test results become available, allowing for a more refined and accurate prediction. The blood tests used in the second stage give a comprehensive assessment of critical physiological systems, providing key indicators of the patient’s condition. Haemoglobin measures the blood’s oxygen-carrying capacity, with low levels indicating anaemia or acute blood loss, both critical in emergency settings. C-Reactive Protein (CRP) serves as a marker of inflammation or infection, aiding in the diagnosis of bacterial infections, sepsis, or inflammatory conditions. Troponin T is a specific indicator of heart damage, essential for diagnosing acute myocardial infarction in patients with chest pain. Glucose (Random Serum) levels assess blood sugar, crucial for identifying hyperglycaemia, often associated with diabetes, or hypoglycaemia, which can cause altered mental states. Platelets are essential for blood clotting, with low counts signalling bleeding risks and high counts possibly indicating inflammatory conditions or cancers. White Blood Cell Count (WBC) reveals immune activity, with elevated levels pointing to infection, inflammation, or stress, while low levels may suggest immune suppression or severe infection. The Estimated Glomerular Filtration Rate (eGFR) reflects kidney function, and a decreased rate indicates impaired renal function, critical for managing fluids and medications. Lastly, Red Cell Distribution Width (RDW) provides insight into the variation in red blood cell size, aiding in diagnosing anaemia and other blood disorders that may complicate patient management in emergency situations. In an emergency setting these markers enable clinicians to rapidly evaluate the severity of acute conditions and guide treatment decisions effectively.

The data used in this study spans a six-year period from January 2017 to December 2022 and encompasses a comprehensive review of all ED visits across Malta, critical for developing predictive models aimed at enhancing ED efficiency and patient care outcomes. The scope of the study was primarily focused on developing and evaluating predictive models; however, the extensive dataset also provided valuable insights beyond model predictions. Key aspects analysed include gender and age distribution, regional variations, admission rates, hospital stay durations, and blood test results, all of which offer a deeper understanding of trends in healthcare utilisation and patient outcomes over time.

Table 2 summarises these key aspects of the dataset analysis. It highlights that gender distribution may influence admission rates and hospital stays. Age groups, especially older patients, tend to have longer hospital stays and higher admission probabilities. There are regional variations that could affect admission rates and specific health concerns. About 31.2% of patients were admitted, with factors such as age, gender, and lab results playing a role in admission likelihood. Hospital stays durations vary widely and are potentially influenced by both demographics and lab results. Abnormal lab results tend to be associated with higher admit rates and longer stays. Admissions also vary seasonally, with peaks in certain seasons like winter, while the dataset’s multi-year span provides an opportunity to analyse trends in healthcare burden over time. More detailed data and comprehensive analysis can be found in Appendix 1.

**Table 2:** High level data interpretation

Aspect	Findings
Gender Distribution	Data includes male and female patients; potential differences in admission rates and stay durations between genders
Age Groups	Age distribution may affect hospital stay durations and admission likelihood, especially in older patients
Geographic Variation (Region)	Regional differences may affect admission rates and specific health concerns across different geographic areas
Admission (Admitted vs Non-Admitted)	31.2% of patients were admitted; factors like age, gender, and blood lab results may affect admission likelihood
Hospital Stay Duration	Hospital stays durations vary widely; blood lab results and demographics may be predictors of longer stays
Blood Test Results (e.g., Haemoglobin, Creatinine, etc.)	Abnormal blood lab results likely correlate with higher admit rates and longer hospital stays
Seasonal Trends	Seasonal variation in admissions, with potential peaks during certain seasons (e.g., winter)
Yearly Changes	Data spans multiple years: analysis could reveal trends in admission rates and healthcare burden

### Predicting patient prioritisation

This section focuses on the process of predicting patient prioritisation. The comparison of model performance between Stage 1 and Stage 2 reveals several key differences as outlined in Table 3.

**Table 3:** Predicting patient prioritisation

Aspect	Stage 1	Stage 2
**Class Distribution (Post-SMOTE)**		
HIGH	325,072	325,072
LOW	325,072	325,072
**Model Performance Metrics**		
Precision (HIGH)	0.70	0.72
Recall (HIGH)	0.59	0.59
F1-Score (HIGH)	0.64	0.65
Precision (LOW)	0.77	0.78
Recall (LOW)	0.85	0.86
F1-Score (LOW)	0.81	0.82
**Overall Accuracy**	**0.75**	**0.76**
Macro Average (Precision)	0.74	0.75
Macro Average (Recall)	0.72	0.73
Macro Average (F1-Score)	0.72	0.73
**Top Features by Importance**		
1. Sub_Category	0.599668	0.280758
2. Complaint_Category	0.245478	0.147617
3. Imaging done at ED	0.077076	0.010940
4. Entry_Method	0.052218	0.034253
5. Part_of_Day	0.008545	0.014772
6. Region	0.007345	0.014055
7. Age_Group	0.004123	0.007176
8. Season	0.003199	0.005793
9. Gender	0.002348	0.002989
10. Estimated_GFR_Serum_Result	N/A	0.192580
11. Haemoglobin_Result	N/A	0.132402
12. White_Blood_Cell_Count_Result	N/A	0.103781
13. Troponin_T_Result	N/A	0.029050
14. Red_Cell_Distribution_Width_Result	N/A	0.012869
15. C_Reactive_Protein_Result	N/A	0.004270
16. Glucose_Random_Serum_Result	N/A	0.003620
17. Platelets_Result	N/A	0.003075

In terms of precision for the “HIGH” class, Stage 1 achieved a precision of 0.70, while Stage 2 showed a slight improvement with a precision of 0.72. This enhancement indicates that Stage 2 was better at correctly identifying true “HIGH” cases when making positive predictions. For recall in the “HIGH” class, Stage 1 achieved 0.59, which remained the same in Stage 2, suggesting that the modifications introduced in Stage 2 did not significantly impact the model's ability to correctly identify instances in this class. Similarly, the F1-score for the “HIGH” class improved slightly from 0.64 in Stage 1 to 0.65 in Stage 2, reflecting a marginal enhancement in the balance between precision and recall.

For the “LOW” class, Stage 1 had a precision of 0.77, while Stage 2 showed a marginal increase to 0.78, indicating a slightly improved ability to correctly predict true “LOW” cases. The recall for the “LOW” class was 0.85 in Stage 1, which increased to 0.86 in Stage 2, enhancing the model’s sensitivity to identifying “LOW” instances. The F1-score for the “LOW” class also rose from 0.81 in Stage 1 to 0.82 in Stage 2, highlighting an overall better performance with more balanced precision and recall.

The overall accuracy of the model increased from 75% in Stage 1 to 76% in Stage 2, suggesting that the extended dataset contributed to a more accurate classification.

When examining the macro averages, Stage 1 had a precision of 0.74, which improved to 0.75 in Stage 2. Similarly, recall increased from 0.72 in Stage 1 to 0.73 in Stage 2, and the F1-score rose from 0.72 to 0.73. These improvements in macro averages indicate that Stage 2 offered a more balanced performance across both the “HIGH” and “LOW” classes.

Stage 2 outperforms Stage 1 in most key metrics, including precision, recall, F1-scores, and overall accuracy, particularly in the classification of the “HIGH” and “LOW” categories. This indicates that the extended dataset in Stage 2 provided additional valuable information that slightly enhanced the model’s predictive capabilities. These results suggest that the model is better at correctly identifying and balancing predictions for “LOW” instances compared to “HIGH” ones, where recall improvements were limited.

**Fig. 4:**
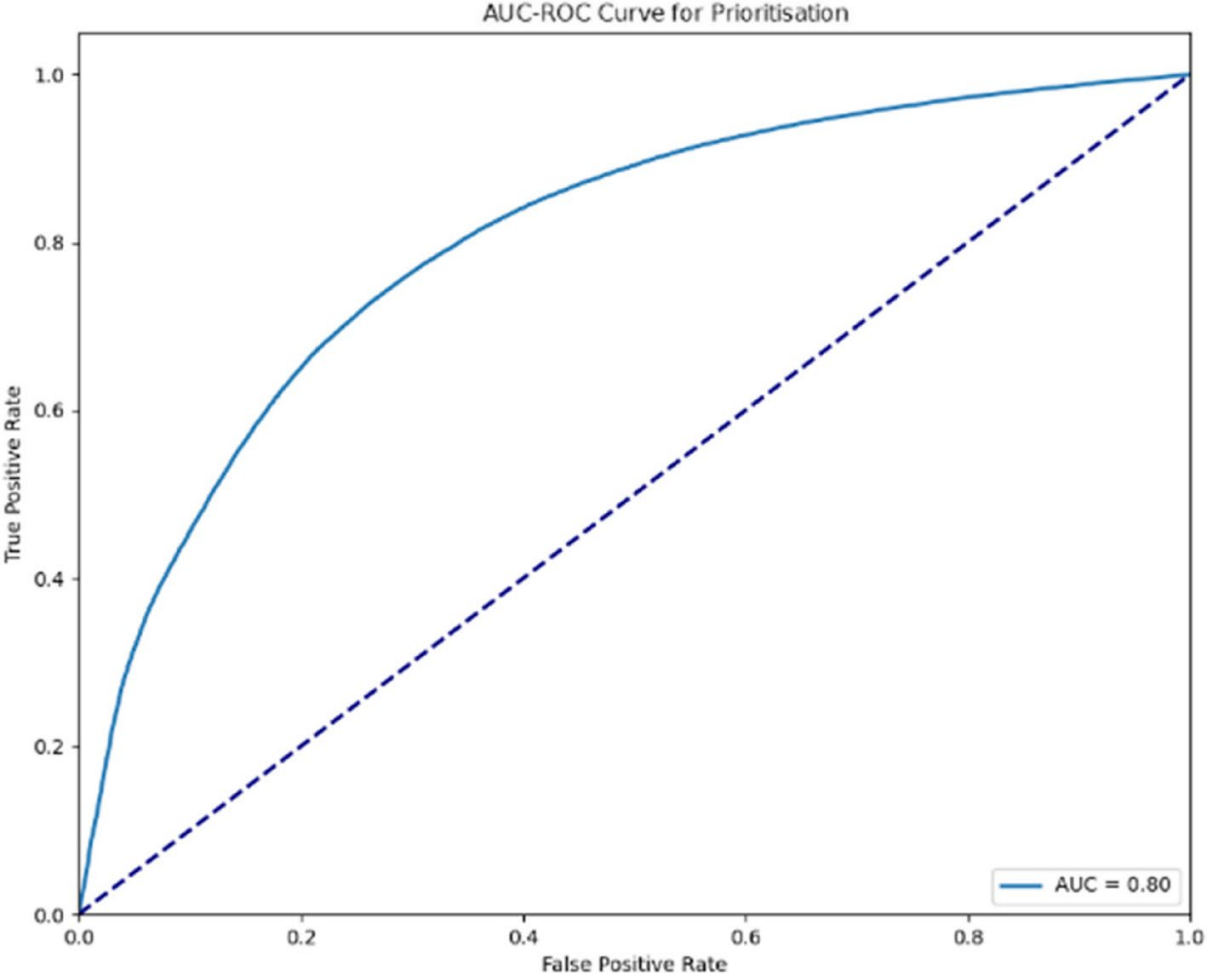
AUC-ROC curve for prioritisation (Stage 1)

**Fig. 5:**
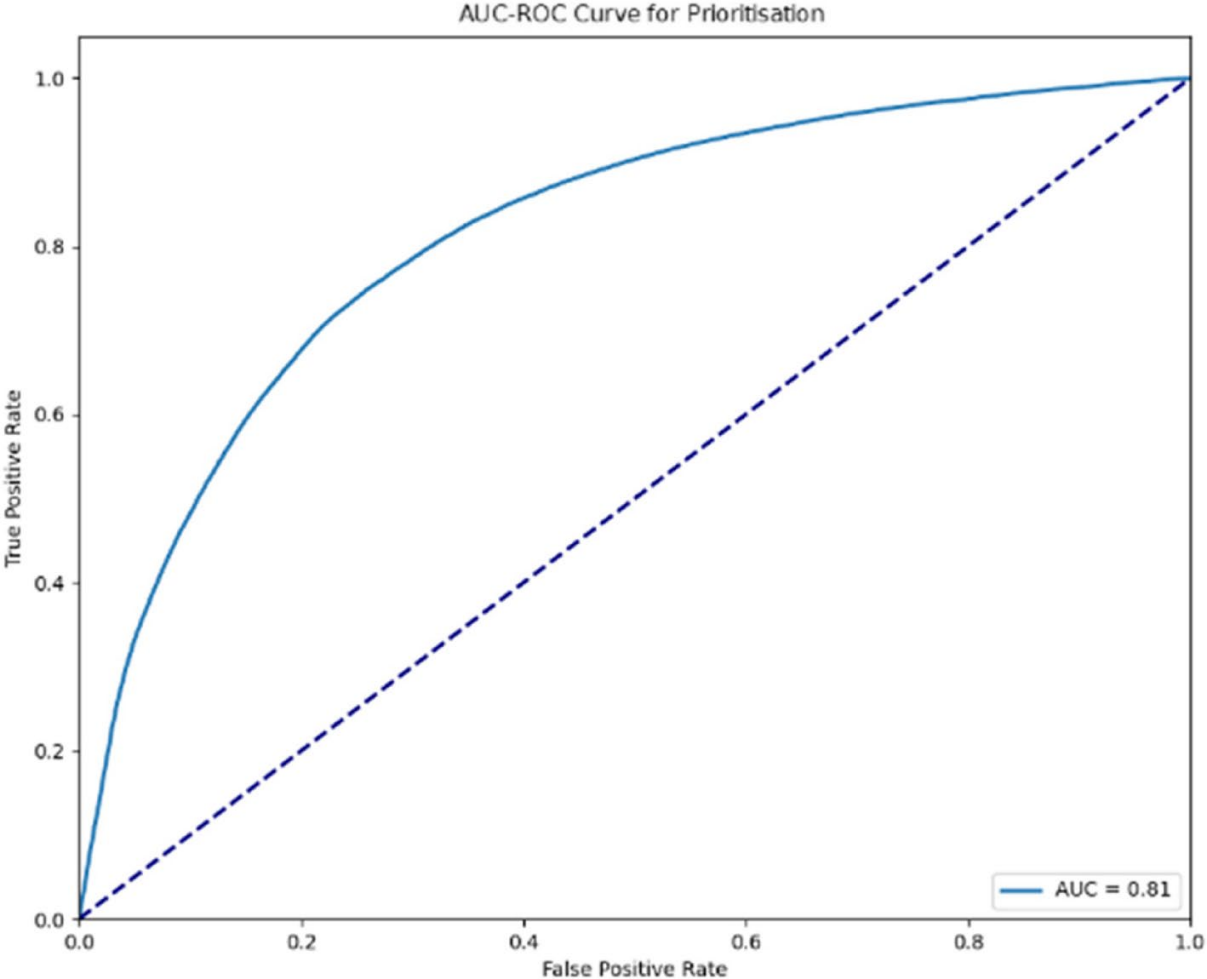
AUC-ROC curve for prioritisation (Stage 2)

The AUC-ROC curves indicated in Figs. 4 and 5 illustrate the performance of the predictive model for prioritisation across two stages of evaluation. The first curve (AUC = 0.80) and the second (AUC = 0.81) both indicate a strong ability of the model to distinguish between the classes. An AUC value closer to 1 suggests better performance, with the incremental improvement between the stages demonstrating the refinement and optimisation of the model. These curves provide visual evidence of the model’s effectiveness in prioritising cases accurately.

**Fig. 6:**
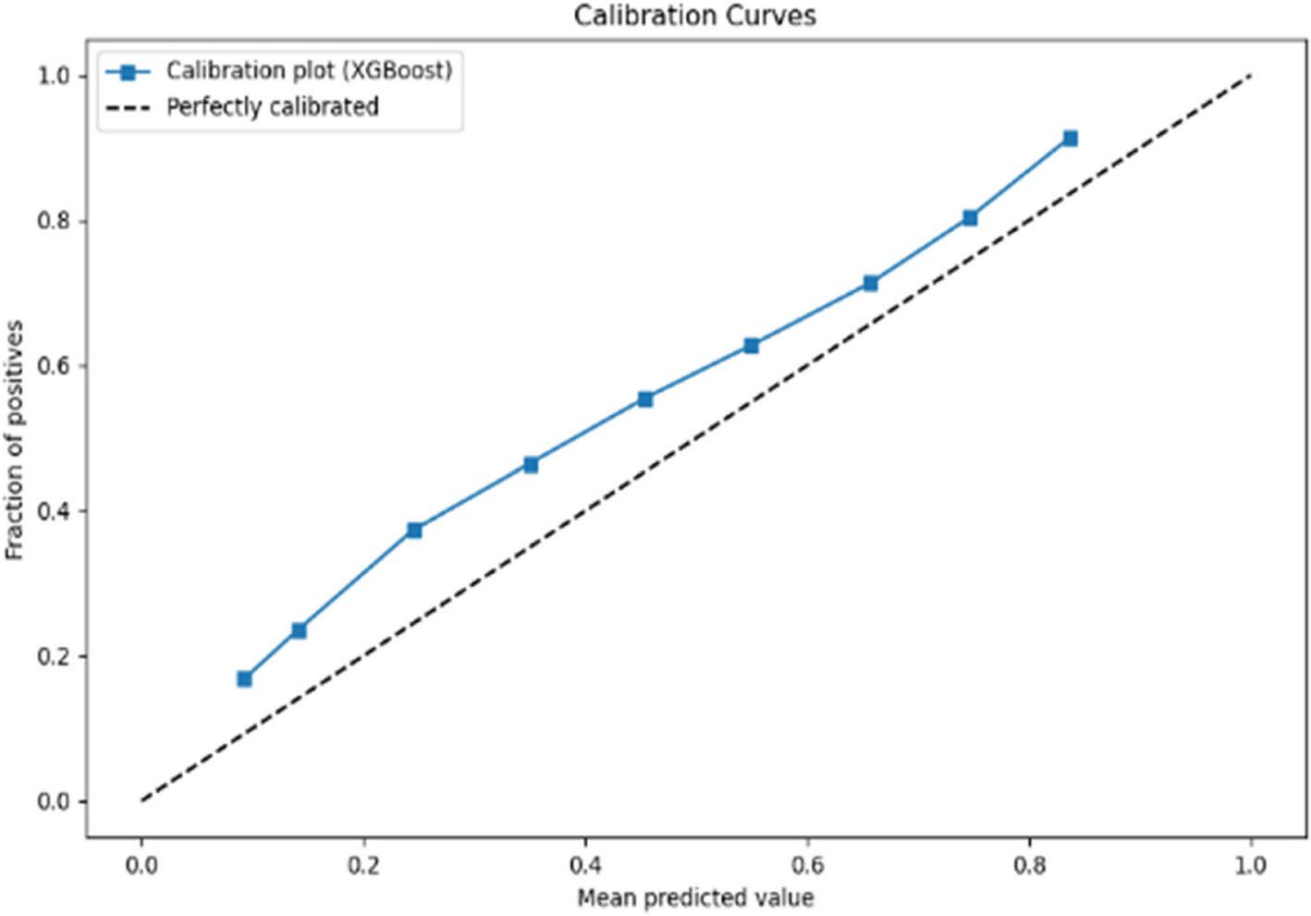
Calibration curve for prioritisation (Stage 1)

**Fig. 7:**
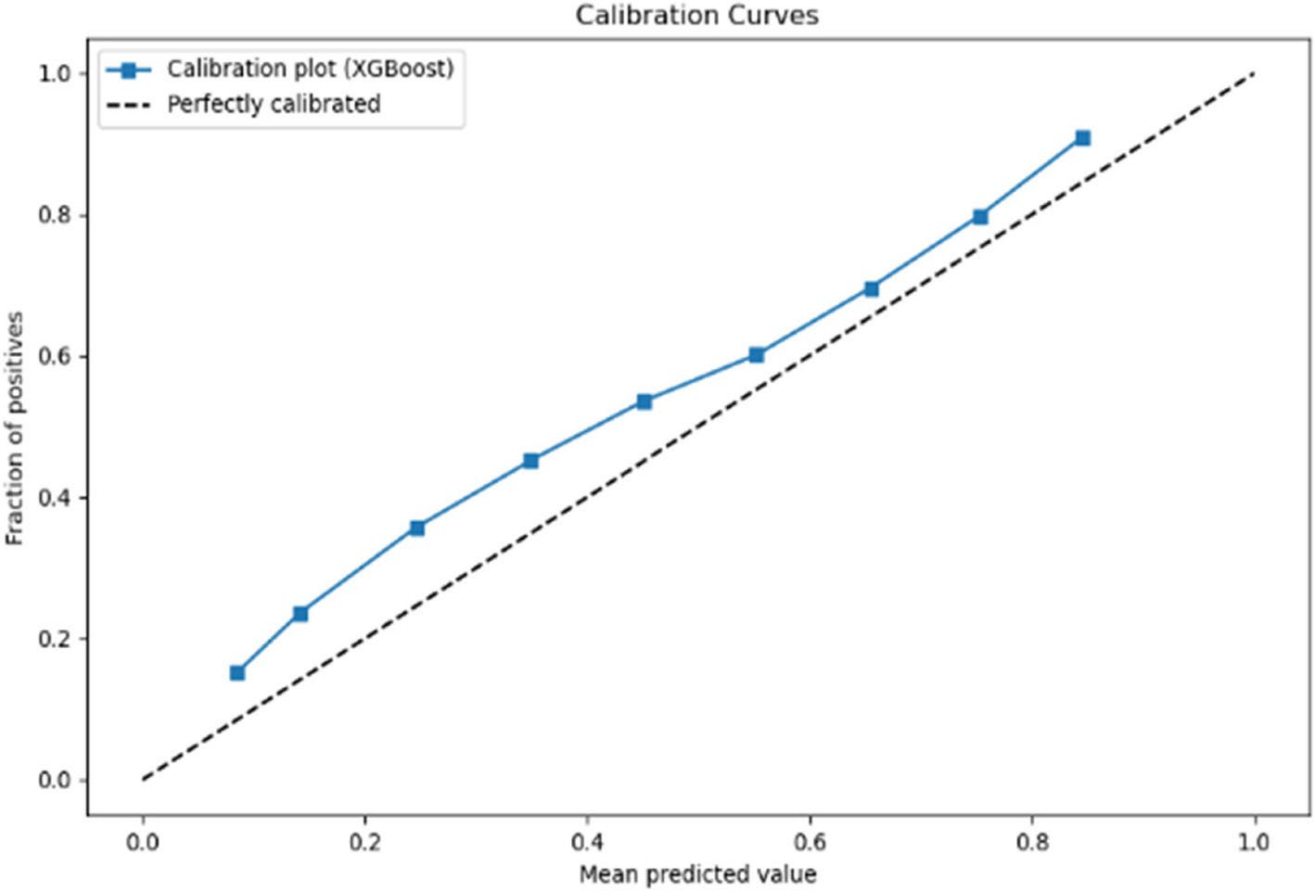
Calibration curve for Prioritisation (Stage 2)

The calibration curves displayed in Figs. 6 and 7 respectively evaluate the accuracy of the predictive model’s probability estimates. The curves show a similar outcome with a progressive increase in the fraction of positives as the mean predicted value rises, illustrating the model’s tendency to underestimate the probability of positives at lower predicted probabilities and slightly overestimate as probabilities increase. While the ideal model would align perfectly with the dashed line representing perfect calibration, this curve reveals that the model is reasonably well-calibrated but could benefit from adjustments to align more closely with the diagonal.

In conclusion, both Stage 1 and Stage 2 highlight the shifting importance of various features in driving model performance. In Stage 1, Sub_Category and Complaint_Category are the dominant features, while in Stage 2, their importance decreases as clinical lab results such as Estimated_GFR_Serum_Result, Haemoglobin_Result, and White_Blood_Cell_Count_Result gain prominence. This shift in Stage 2 suggests a greater reliance on clinical data to enhance prediction accuracy. While demographic features like Age_Group, Gender, and Region maintain relatively low importance across both stages, the overall evolution in feature importance reflects the model’s improved ability to integrate both categorical and clinical data for better prediction outcomes.

### Predicting patient admission

This section focuses on the process of predicting patient admission as outlined in Table 4. The comparison of model performance between Stage 1 and Stage 2 reveals several key differences. Both models utilised SMOTE to balance the class distribution, resulting in 359,244 instances for both the “Admitted” and “Not Admitted” categories, ensuring that neither model was biased toward either class.

**Table 4:** Predicting patient admission

Aspect	Stage 1	Stage 2
**Class Distribution (Post-SMOTE)**		
Admitted	359,244	359,244
Not Admitted	359,244	359,244
**Model Performance Metrics**		
Precision (Not Admitted)	0.88	0.90
Recall (Not Admitted)	0.83	0.84
F1-Score (Not Admitted)	0.85	0.87
Precision (Admitted)	0.66	0.69
Recall (Admitted)	0.74	0.79
F1-Score (Admitted)	0.70	0.74
**Overall Accuracy**	**0.80**	**0.82**
Macro Average (Precision)	0.77	0.79
Macro Average (Recall)	0.78	0.81
Macro Average (F1-Score)	0.77	0.80
**Top Features by Importance**		
1. Main_Complaint_Category	0.366452	0.098703
2. Sub_Category	0.340276	0.166274
3. Imaging done at ED	0.194817	0.022586
4. Entry_Method	0.056628	0.013569
5. Age_Group	0.014497	0.010499
6. Part_of_Day	0.010952	0.016365
7. Region	0.010224	0.012885
8. Season	0.003579	0.004886
9. Gender	0.002577	0.002411
10. Haemoglobin_Result	N/A	0.299525
11. Red_Cell_Distribution_Width_Result	N/A	0.200899
12. White_Blood_Cell_Count_Result	N/A	0.120217
13. Troponin_T_Result	N/A	0.013900
14. Estimated_GFR_Serum_Result	N/A	0.006094
15. C_Reactive_Protein_Result	N/A	0.006083
16. Glucose_Random_Serum_Result	N/A	0.003013
17. Platelets_Result	N/A	0.002092

In terms of precision for the “Not Admitted” class, Stage 1 achieved a precision accuracy of 0.88, while Stage 2 showed a notable improvement with a precision of 0.90. This enhancement indicates that Stage 2 was better at correctly identifying true “Not Admitted” cases when making negative predictions. For recall in the “Not Admitted” class, Stage 1 achieved 0.83, which increased slightly to 0.84 in Stage 2, suggesting that the extended dataset in Stage 2 did affect slightly the model’s ability to identify actual “Not Admitted” cases. Similarly, the F1-score for the “Not Admitted” class improved from 0.85 in Stage 1 to 0.87 in Stage 2, reflecting a more balanced performance between precision and recall.

For the “Admitted” class, Stage 1 had a precision of 0.66, while Stage 2 had a value of 0.69, indicating a slightly improved ability to correctly predict true “Admitted” cases. The recall for the “Admitted” class was 0.74 in Stage 1, which increased to 0.79 in Stage 2, enhancing the model’s sensitivity to identifying “Admitted” instances. The F1-score for the “Admitted” class also rose from 0.70 in Stage 1 to 0.74 in Stage 2, highlighting an overall better performance with more balanced precision and recall.

The overall accuracy of the model increased from 80% in Stage 1 to 82% in Stage 2, suggesting that the extended dataset contributed to a more accurate classification. When examining the macro averages, Stage 1 had a precision of 0.77, which improved to 0.79 in Stage 2. Similarly, recall increased from 0.78 in Stage 1 to 0.81 in Stage 2, and the F1-score rose from 0.77 to 0.80. These improvements in macro averages indicate that Stage 2 offered a more balanced performance across both the “Admitted” and “Not Admitted” classes.

Stage 2 outperforms Stage 1 in all key metrics, including precision, recall, F1-scores, and overall accuracy, particularly in the classification of the “Admitted” and “Not Admitted” categories. This indicates that the extended dataset in Stage 2 provided additional valuable information that enhanced the model’s predictive capabilities. Overall, the model is more precise and balanced in identifying “Not Admitted” instances, while for “Admitted” cases, the model shows improvement, particularly in recall, but still lags behind in precision. This indicates that the model is better at correctly identifying “Not Admitted” cases compared to “Admitted” ones.

**Fig. 8:**
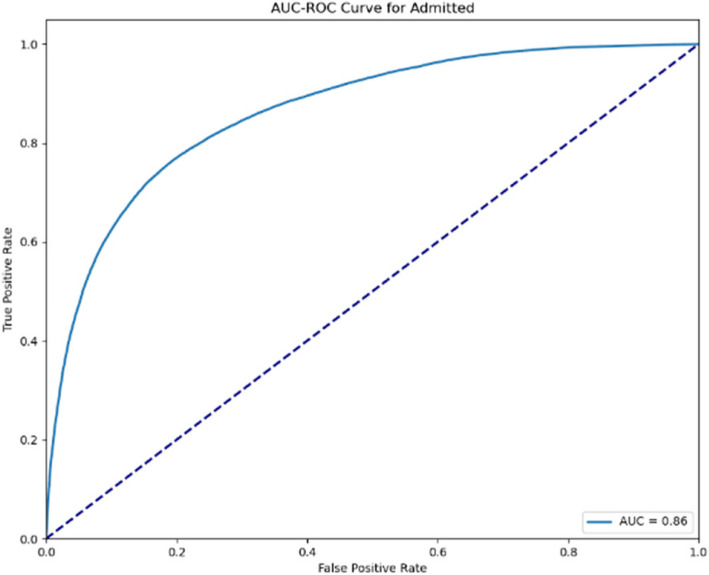
AUC-ROC curve for admission (Stage 1)

**Fig. 9:**
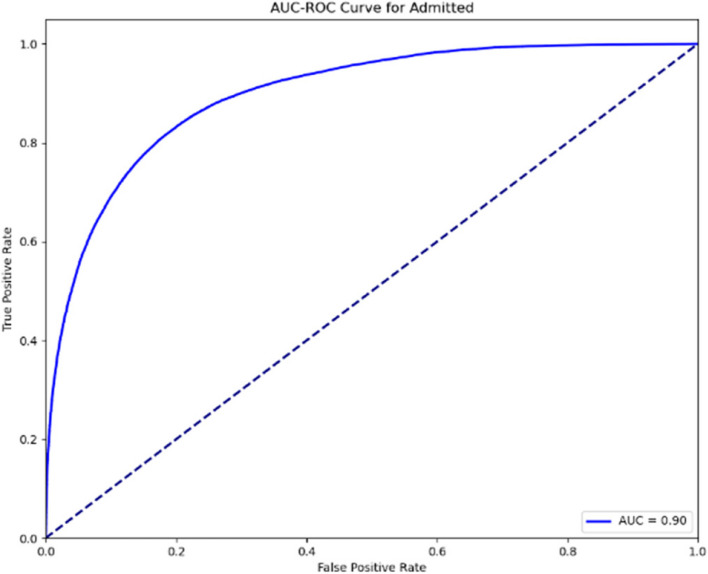
AUC-ROC curve for admission (Stage 2)

The AUC-ROC curves displayed in Figs. 8 and 9 below represent the performance of the predictive model for the “Admitted” category across the two evaluation stages. The first curve (AUC = 0.86) indicates a strong classification ability, while the second (AUC = 0.90) shows further improvement, demonstrating an even higher ability to differentiate between outcomes. The increase in the AUC value reflects the model’s enhancement and refinement, supporting its reliability in predicting admissions accurately.

**Fig. 10:**
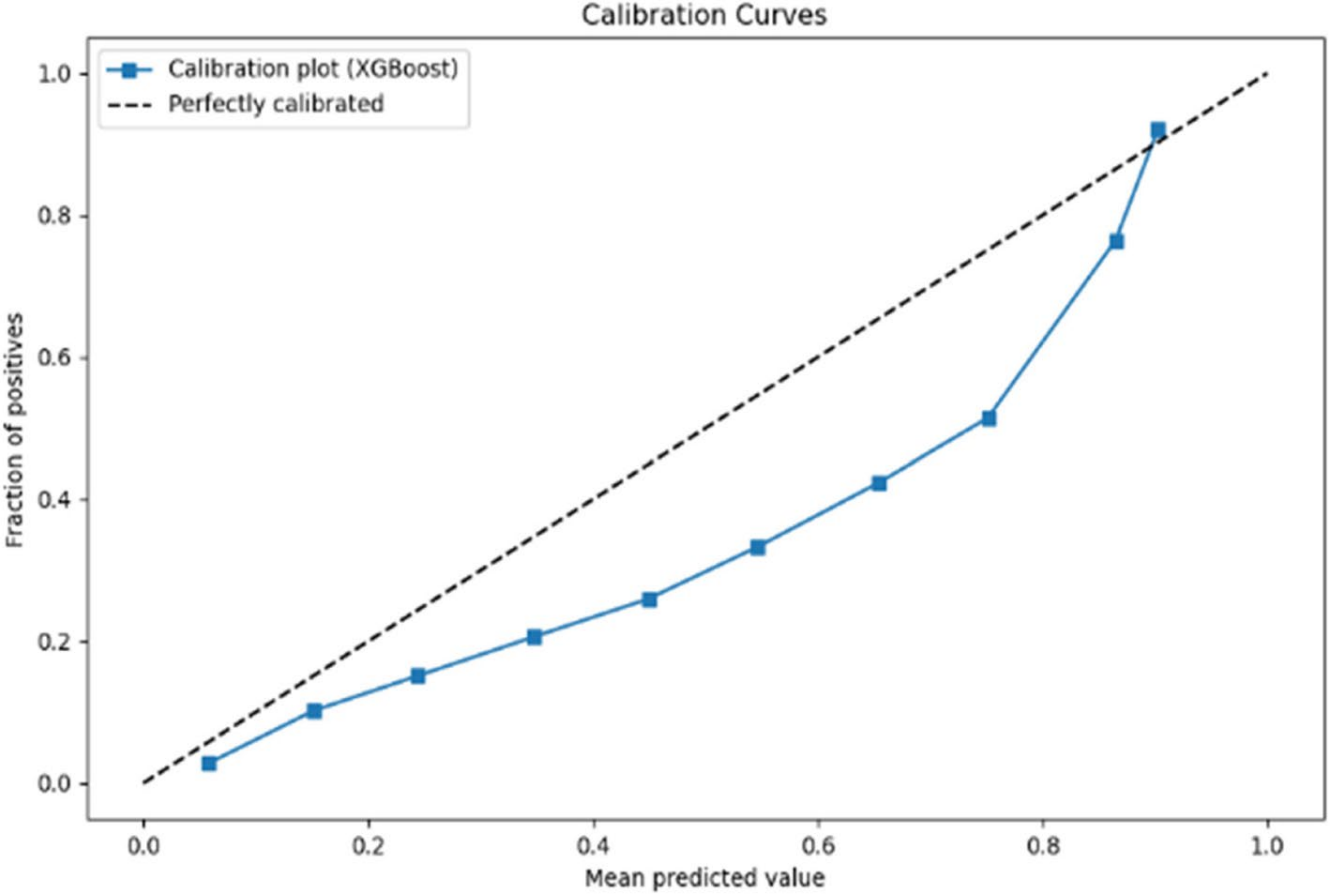
Calibration curve for admission (Stage 1)

**Fig. 11:**
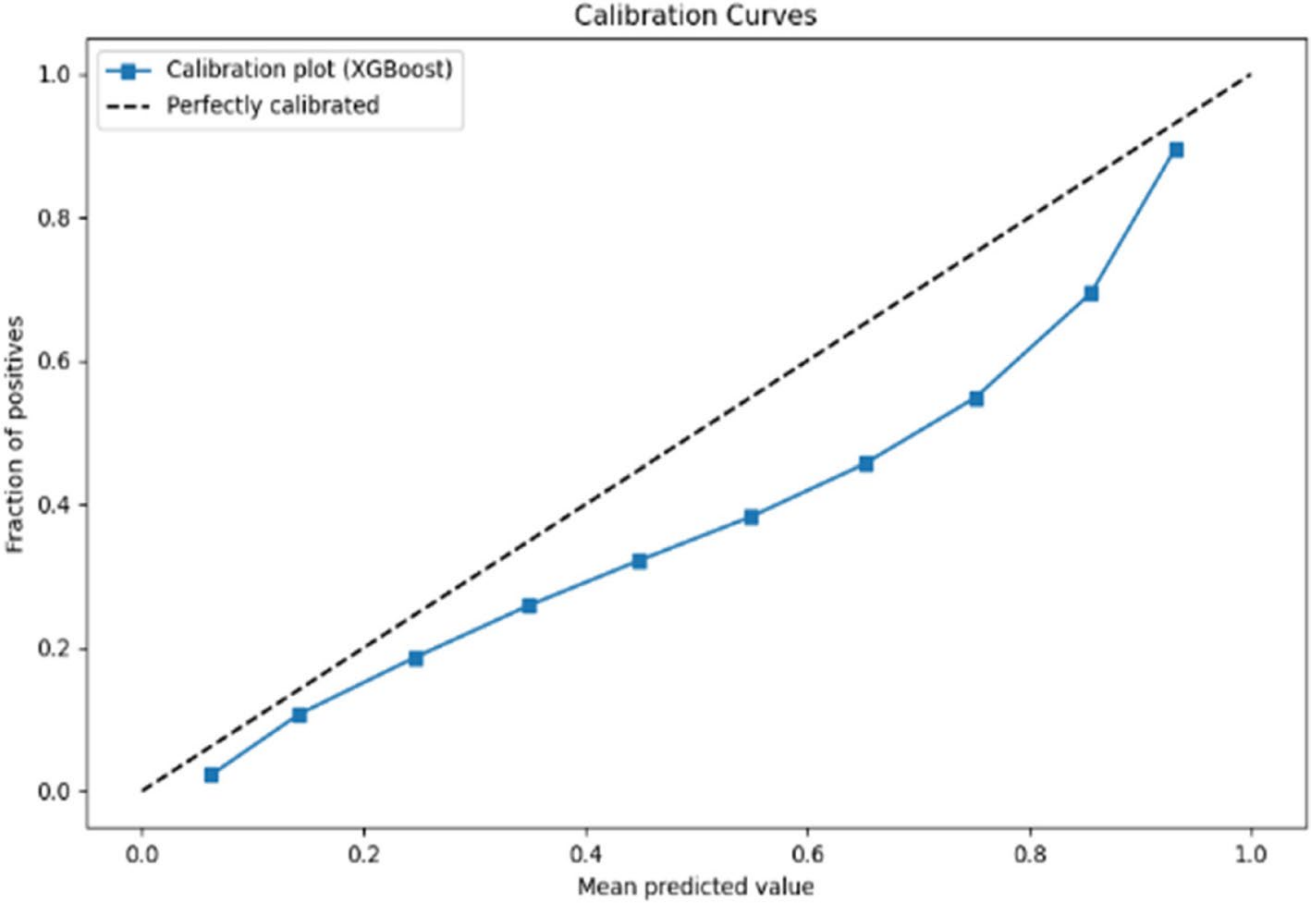
Calibration curve for admission (Stage 2)

The calibration curves for the predictive model, shown across Stage 1 and Stage 2 in Figs. 10 and 11 respectively, reveal its probability estimation accuracy. Both stages exhibit a pattern where the model underestimates positive outcomes at lower probabilities and overestimates at higher probabilities. The curves approach perfect calibration as predicted probabilities increase, particularly in the Stage 2 where the model’s predictions nearly align with actual outcomes at high confidence levels. Despite reasonable overall calibration, the deviation at higher probabilities suggests potential for refinement, such as adjusting probability thresholds or applying calibration techniques to enhance model accuracy.

In conclusion, both Stage 1 and Stage 2 of the model emphasise the importance of clinical and demographic features in driving predictive performance. While Main_Complaint_Category and Sub_Category remain key features in both stages, their importance shifts between Stage 1 and Stage 2, with a greater emphasis on lab results such as Haemoglobin_Result and Red_Cell_Distribution_Width_Result in Stage 2. This suggests that Stage 2 places increased weight on clinical laboratory data, enhancing the model’s ability to make more accurate predictions. The model evolves across stages, balancing the contribution of categorical features with lab results, leading to more refined and reliable performance.

### Predicting admitting ward

The structure of the hospital’s admitting wards is complex, encompassing an extensive list of different ward categories. For the purposes of this predictive model, the wards were systematically categorised into two levels: main wards and sub-wards as indicated in Appendix 2.

#### Main category admitting ward

This section focuses on the process of predicting main category admitting ward. The comparison of model performance between Stage 1 and Stage 2 highlights several significant differences as outlined in Table 5.

**Table 5:** Main category admitting ward

Aspect	Stage 1	Stage 2
**Class Distribution (Post-SMOTE)**	Balanced (107,093 instances)	Balanced (107,093 instances)
Cardiology	17,849	17,849
Medicine	17,849	17,849
Obs & Gynae	17,849	17,849
Paediatrics	17,848	17,848
Specialty Care	17,849	17,849
Surgery	17,849	17,849
**Model Performance Metrics**		
Precision (Cardiology)	0.70	0.84
Recall (Cardiology)	0.78	0.85
F1-Score (Cardiology)	0.74	0.85
Precision (Medicine)	0.70	0.78
Recall (Medicine)	0.54	0.74
F1-Score (Medicine)	0.61	0.76
Precision (Obs & Gynae)	0.90	0.93
Recall (Obs & Gynae)	0.97	0.98
F1-Score (Obs & Gynae)	0.93	0.95
Precision (Paediatrics)	0.95	0.96
Recall (Paediatrics)	0.98	0.99
F1-Score (Paediatrics)	0.97	0.97
Precision (Specialty Care)	0.77	0.83
Recall (Specialty Care)	0.85	0.88
F1-Score (Specialty Care)	0.81	0.86
Precision (Surgery)	0.77	0.83
Recall (Surgery)	0.68	0.75
F1-Score (Surgery)	0.72	0.79
Overall Accuracy	0.80	0.86
Macro Average (Precision)	0.80	0.86
Macro Average (Recall)	0.80	0.86
Macro Average (F1-Score)	0.80	0.86
**Top Features by Importance**		
1. Main_Complaint_Category	0.534174	0.474656
2. Sub_Category	0.36959	0.245715
3. Age_Group	0.025675	0.024526
4. Gender	0.019845	0.021517
5. Imaging done at ED	0.016466	0.01809
6. Region	0.013191	0.015512
7. Entry_Method	0.009025	0.009785
8. Part_of_Day	0.006303	0.00744
9. Season	0.005731	0.006541
10. Troponin_T_Result	0	0.090083
11. Estimated_GFR_Serum_Result	0	0.042822
12. C_Reactive_Protein_Result	0	0.011233
13. Glucose_Random_Serum_Result	0	0.009102
14. Red_Cell_Distribution_Width_Result	0	0.007492
15. Platelets_Result	0	0.005589
16. Haemoglobin_Result	0	0.005198
17. White_Blood_Cell_Count_Result	0	0.004699

In terms of precision for the “Medicine” ward, Stage 1 achieved a value of 0.70, while Stage 2 showed an improvement, with a precision of 0.78. This increase suggests that Stage 2 was more effective in correctly identifying true cases for the “Medicine” category. For recall in the “Medicine” class, Stage 1 achieved 0.54, and Stage 2 increased this value to 0.74, indicating that the Stage 2 model had better sensitivity in identifying actual “Medicine” cases. Similarly, the F1-score for “Medicine” improved from 0.61 in Stage 1 to 0.76 in Stage 2, reflecting a more balanced performance between precision and recall.

For the “Surgery” class, Stage 1 had a precision of 0.77, while Stage 2 improved to 0.83, indicating a clear enhancement in correctly predicting true “Surgery” cases. The recall for the “Surgery” class in Stage 1 was 0.68, which increased to 0.75 in Stage 2, further enhancing the model's sensitivity for this category. The F1-score for “Surgery” also rose from 0.72 in Stage 1 to 0.79 in Stage 2, demonstrating improved overall performance.

For the “Paediatrics” and “Obs & Gynae” categories, Stage 1 already demonstrated high precision and recall, with values close to or exceeding 0.95. These strong results were maintained in Stage 2, confirming the model's robustness in identifying patients in these categories.

The overall accuracy of the model increased from 80% in Stage 1 to 86% in Stage 2, indicating that the additional data introduced in Stage 2 contributed to more accurate classifications across all ward categories. Examining the macro averages, Stage 1 had a precision of 0.80, which improved to 0.86 in Stage 2. Similarly, recall increased from 0.80 in Stage 1 to 0.86 in Stage 2, and the F1-score rose from 0.80 to 0.86. These improvements in macro averages suggest that Stage 2 delivered more balanced and reliable predictions across all classes.

Stage 2 consistently outperformed Stage 1 in all key metrics, including precision, recall, F1-scores, and overall accuracy. This improvement is especially pronounced in categories like “Medicine” and “Surgery,” where Stage 1 struggled to achieve strong predictive performance. The extended dataset model in Stage 2 provided valuable information that significantly enhanced its predictive capabilities.

**Fig. 12:**
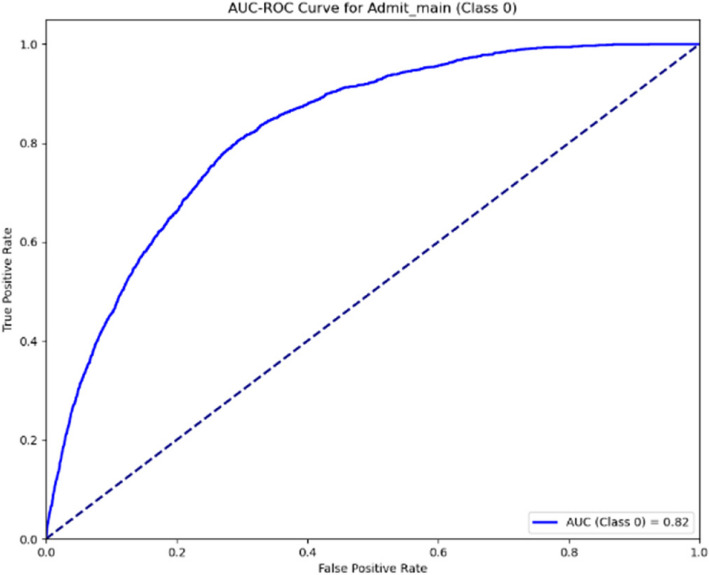
AUC-ROC curve for ward (Stage 1)

**Fig. 13:**
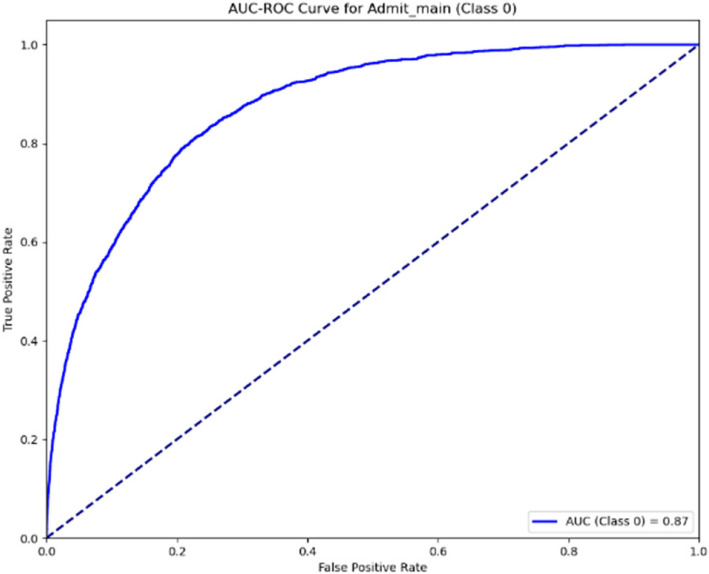
AUC-ROC curve for ward (Stage 2)

The AUC-ROC curves presented for the patient admission outlined in Figs. 12 and 13 illustrate the model’s performance at two stages. The first curve, with an AUC of 0.82, shows the initial classification capability, while the second curve, with an improved AUC of 0.87, demonstrates enhanced discriminatory power. This improvement reflects the model’s refinement, suggesting increased accuracy in predicting outcomes in the later stage.

**Fig. 14:**
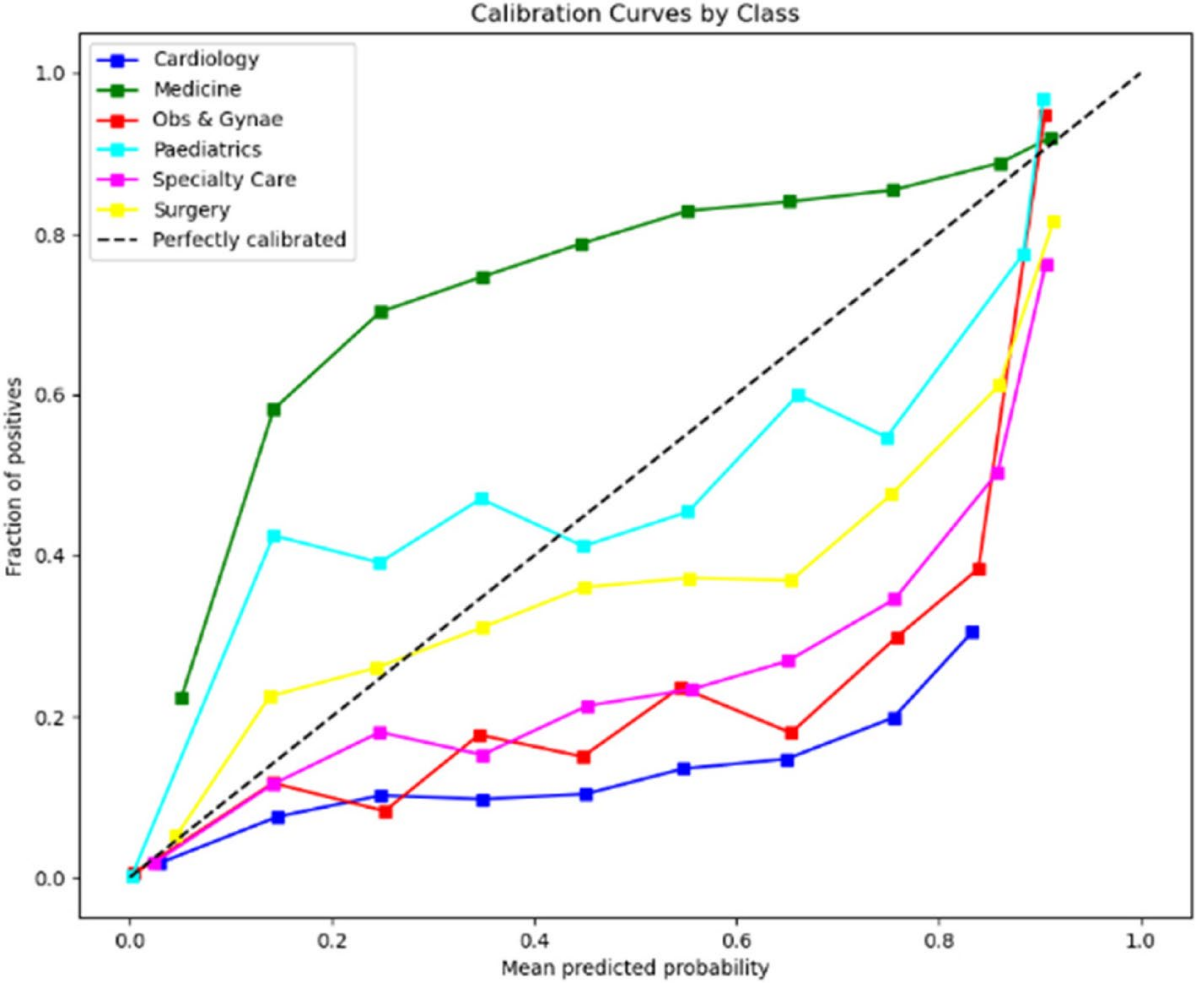
Calibration curve for ward (Stage 1)

**Fig. 15:**
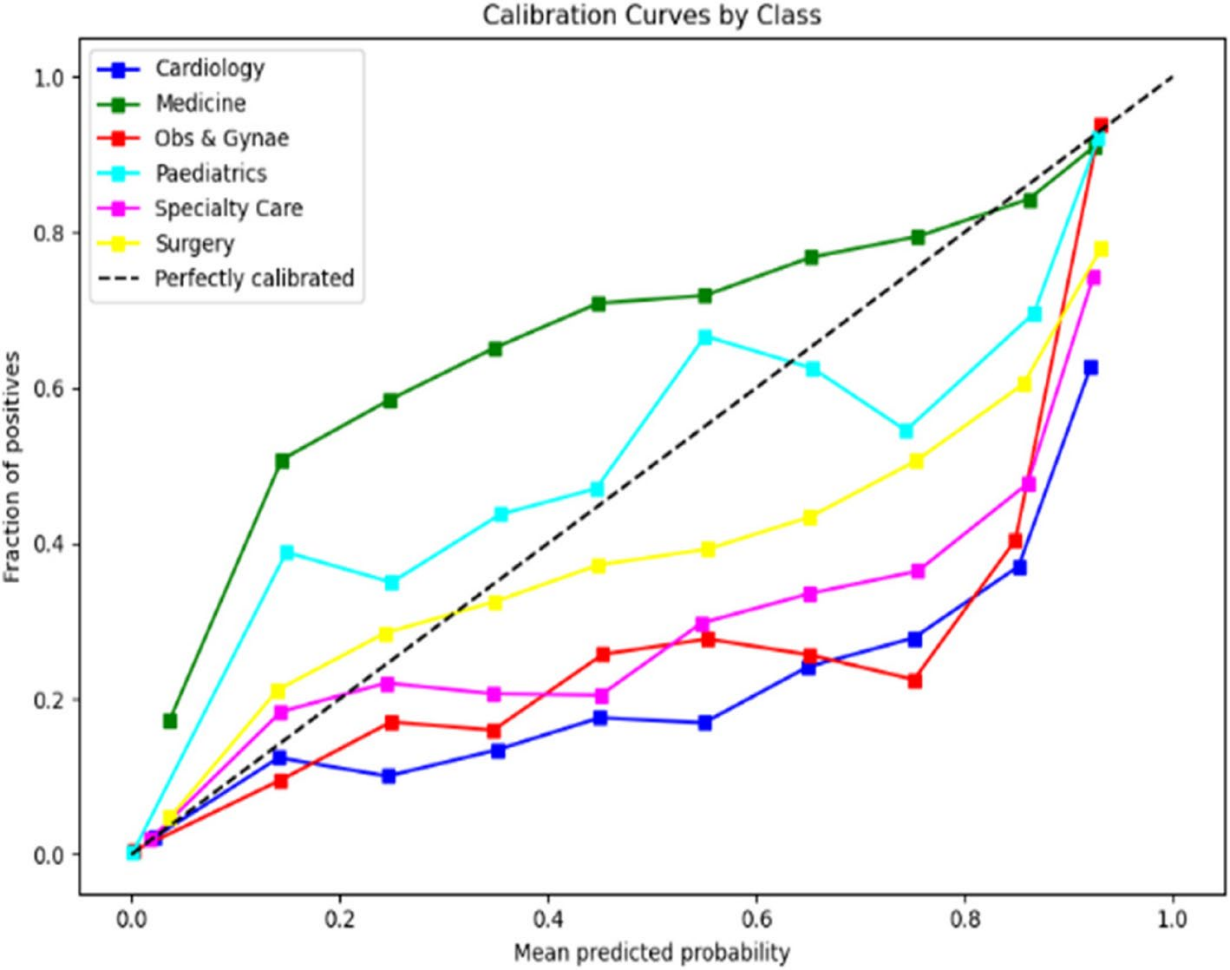
Calibration curve for ward (Stage 2)

In the analysis of calibration curves outlined in Figs. 14 and 15 across two distinct stages of predictive modelling, there is a marked improvement from Stage 1 to Stage 2, which can be attributed to the integration of more comprehensive clinical data. In Stage 1, the calibration curves for various medical specialties such as Cardiology, Medicine, and Paediatrics show a significant deviation from perfect calibration, particularly as the predicted probabilities approach 1, indicating a consistent underprediction of the true probabilities. Conversely, in Stage 2, where more detailed data (likely from further tests and investigations) is incorporated, the calibration curves are noticeably closer to the line of perfect calibration. This is particularly evident in specialties like Specialty Care and Surgery, suggesting a substantial enhancement in the accuracy of the model’s predictions. These observations highlight the critical role of detailed and comprehensive data in enhancing the reliability and utility of predictive models in clinical settings.

In conclusion, both Stage 1 and Stage 2 demonstrate the importance of clinical and demographic features in the model''s predictions, though with noticeable shifts in emphasis. In both stages, Main_Complaint_Category and Sub_Category remain the top features, but their importance decreases slightly in Stage 2. Meanwhile, clinical lab results, such as Troponin_T_Result and Estimated_GFR_Serum_Result, which were not influential in Stage 1, gain significant importance in Stage 2. Demographic features like Age_Group and Gender maintain relatively stable importance across both stages, while features such as Imaging done at ED and Region show slight increases. This progression highlights the model's increasing reliance on clinical data in Stage 2 to improve predictive performance (Table 6).

**Table 6:** Subcategory admitting ward

Aspect	Stage 1	Stage 2
**Class Distribution (Post-SMOTE)**	Balanced (113,566 instances) 3,154 / 3,155 instances per ward	Balanced (113,566 instances) 3,154 / 3,155 instances per ward
**Model Performance Metrics**		
Precision (Macro Average)	0.66	0.73
Recall (Macro Average)	0.69	0.75
F1-Score (Macro Average)	0.67	0.74
Precision (Weighted Average)	0.66	0.73
Recall (Weighted Average)	0.69	0.75
F1-Score (Weighted Average)	0.67	0.74
Overall Accuracy	0.69	0.75
**Top Features by Importance**		
1. Main_Complaint_Category	0.534174	0.474656
2. Sub_Category	0.36959	0.245715
3. Age_Group	0.025675	0.024526
4. Gender	0.019845	0.021517
5. Imaging done at ED	0.016466	0.01809
6. Region	0.013191	0.015512
7. Entry_Method	0.009025	0.009785
8. Part_of_Day	0.006303	0.00744
9. Season	0.005731	0.006541
10. Troponin_T_Result	0	0.090083
11. Estimated_GFR_Serum_Result	0	0.042822
12. C_Reactive_Protein_Result	0	0.019068
13. Glucose_Random_Serum_Result	0	0.009102
14. Red_Cell_Distribution_Width_Result	0	0.007492
15. Platelets_Result	0	0.005589
16. Haemoglobin_Result	0	0.005198
17. White_Blood_Cell_Count_Result	0	0.004699

#### Subcategory admitting ward

This section focuses on the process of predicting subcategory admitting ward.

In Stage 1, the overall model accuracy for subcategory wards was 69%. While categories such as “Accident & Emergency” demonstrated excellent performance with high precision and recall, subcategories within “Medicine,” such as “Medicine/Acute,” “Medicine/Diabetes/Endo,” and “Medicine/Respiratory,” exhibited lower predictive performance. For instance, “Medicine/Acute” achieved a precision of 0.14 and recall of 0.07, reflecting poor sensitivity in identifying true positive cases. Similarly, “Medicine/Nephrology” and “Medicine/Respiratory” also had low F1-scores, highlighting significant imbalances between precision and recall.

Stage 2 showed substantial progress, with overall model accuracy rising to 75%. Precision and recall improved across most subcategory wards. For example, the “Cardiology” subcategory saw its precision increase from 0.25 in Stage 1 to 0.42 in Stage 2, with a corresponding recall increase from 0.56 to 0.70. This improvement reflects the model's enhanced ability to correctly classify 'Cardiology' cases, reducing misclassification rates.

Subcategories within the “Medicine” category also experienced improvements. “Medicine/Geriatrics,” for example, maintained a high recall, improving from 0.96 in Stage 1 to 0.99 in Stage 2. While precision remained modest in certain 'Medicine' subcategories, the overall F1-scores improved, indicating more balanced model predictions.

In particular, the “Medicine/Acute” subcategory, despite its precision remaining low (0.17 in Stage 2), showed a slight improvement in recall, suggesting a better ability to capture true cases in this category. Likewise, “Medicine/Respiratory” saw precision increase from 0.19 to 0.22, and recall improved as well, resulting in a more balanced performance. In the “Surgery General” subcategory, precision increased from 0.31 to 0.38, and recall rose from 0.38 to 0.44 in Stage 2, reflecting better performance in predicting surgical cases.

The macro averages for precision, recall, and F1-score across all subcategories improved from 0.66 in Stage 1 to 0.73 in Stage 2. This shows that the Stage 2 model provided more consistent and reliable predictions across a wide range of subcategories. The inclusion of features such as blood test results and entry methods in Stage 2 contributed to these improvements, allowing the model to capture more complex patterns and enhance its predictive power.

Stage 2 demonstrated significant improvements in predictive performance, particularly within the “Medicine” and “Surgery” categories. The refinements in Stage 2, including a more comprehensive feature set, led to better sensitivity and precision, making the model more effective for aiding decision-making in hospital triage and admissions. These findings underscore the value of incorporating domain-specific features and advanced techniques like SMOTE to achieve balanced and accurate predictions in healthcare predictive modelling.

**Fig. 16:**
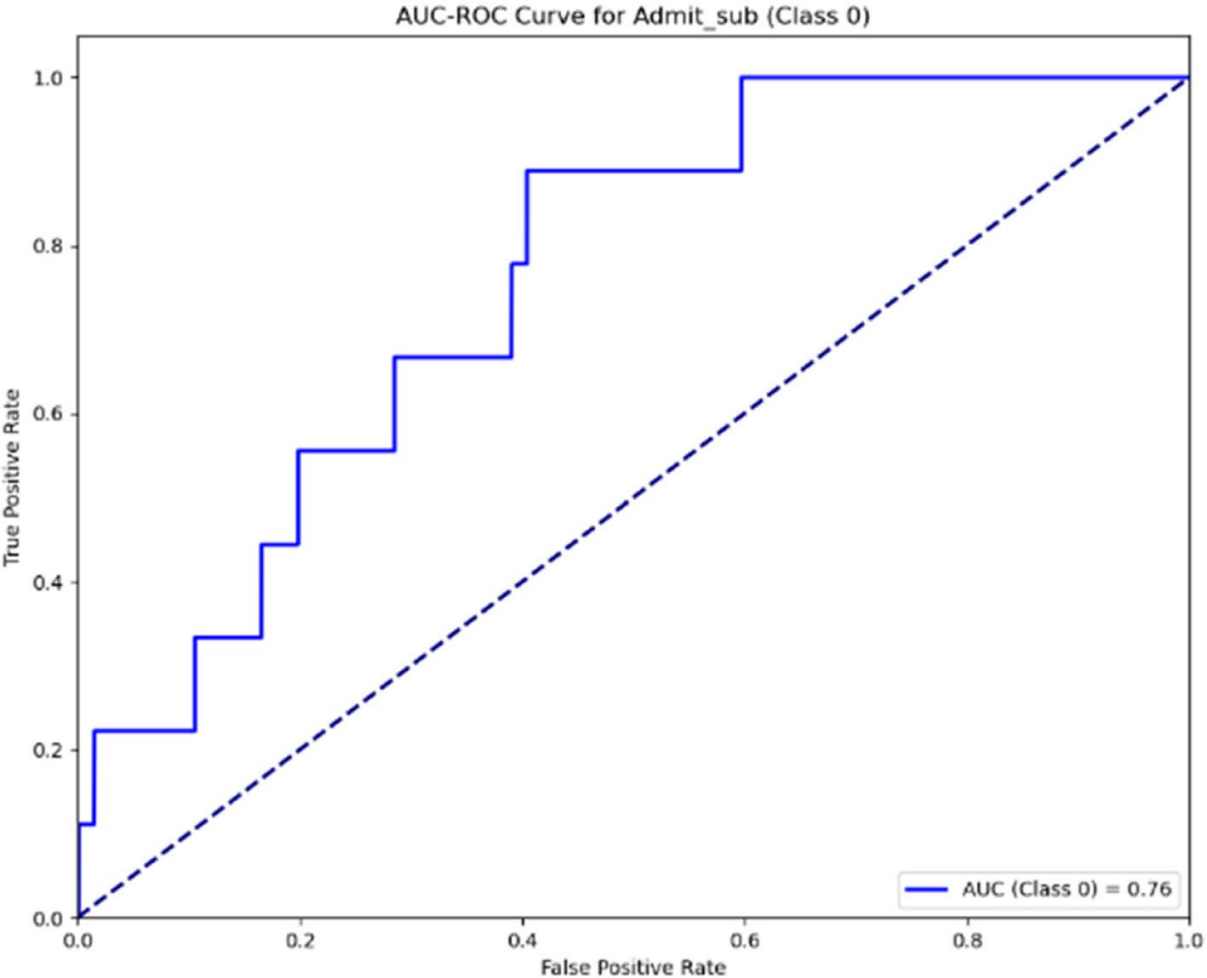
AUC-ROC curve for sub-ward (Stage 1)

**Fig. 17:**
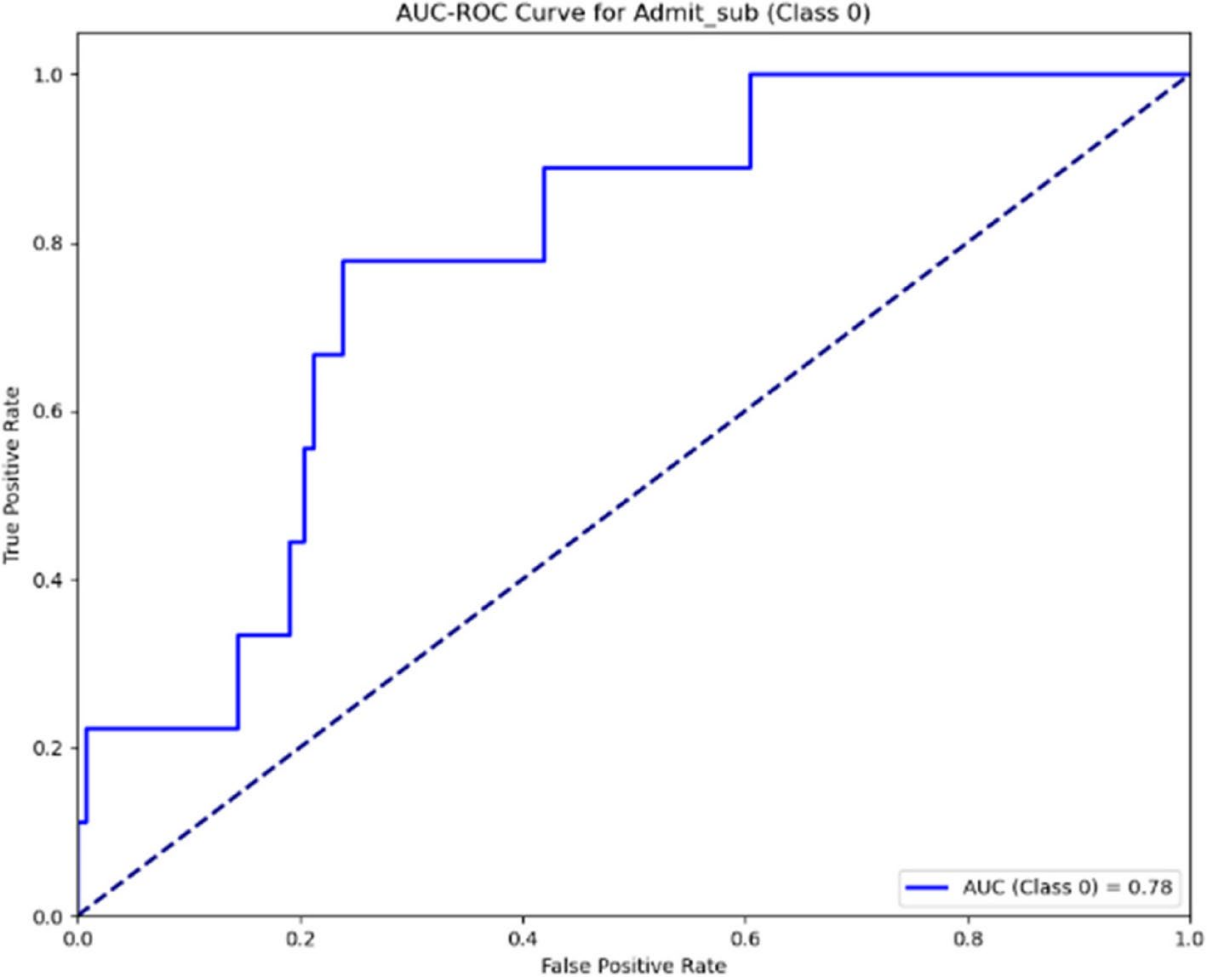
AUC-ROC curve for sub-ward (Stage 2)

The AUC-ROC curves for the Sub-Ward indicated in Figs. 16 and 17 show the model’s performance across two stages. In the first stage, the AUC is 0.76, indicating a moderate level of classification accuracy. In the second stage, an improvement is seen with the AUC rising to 0.78, suggesting a slight enhancement in the model’s ability to differentiate between the classes. This progression highlights ongoing efforts to optimise the model’s predictive performance.

**Fig. 18:**
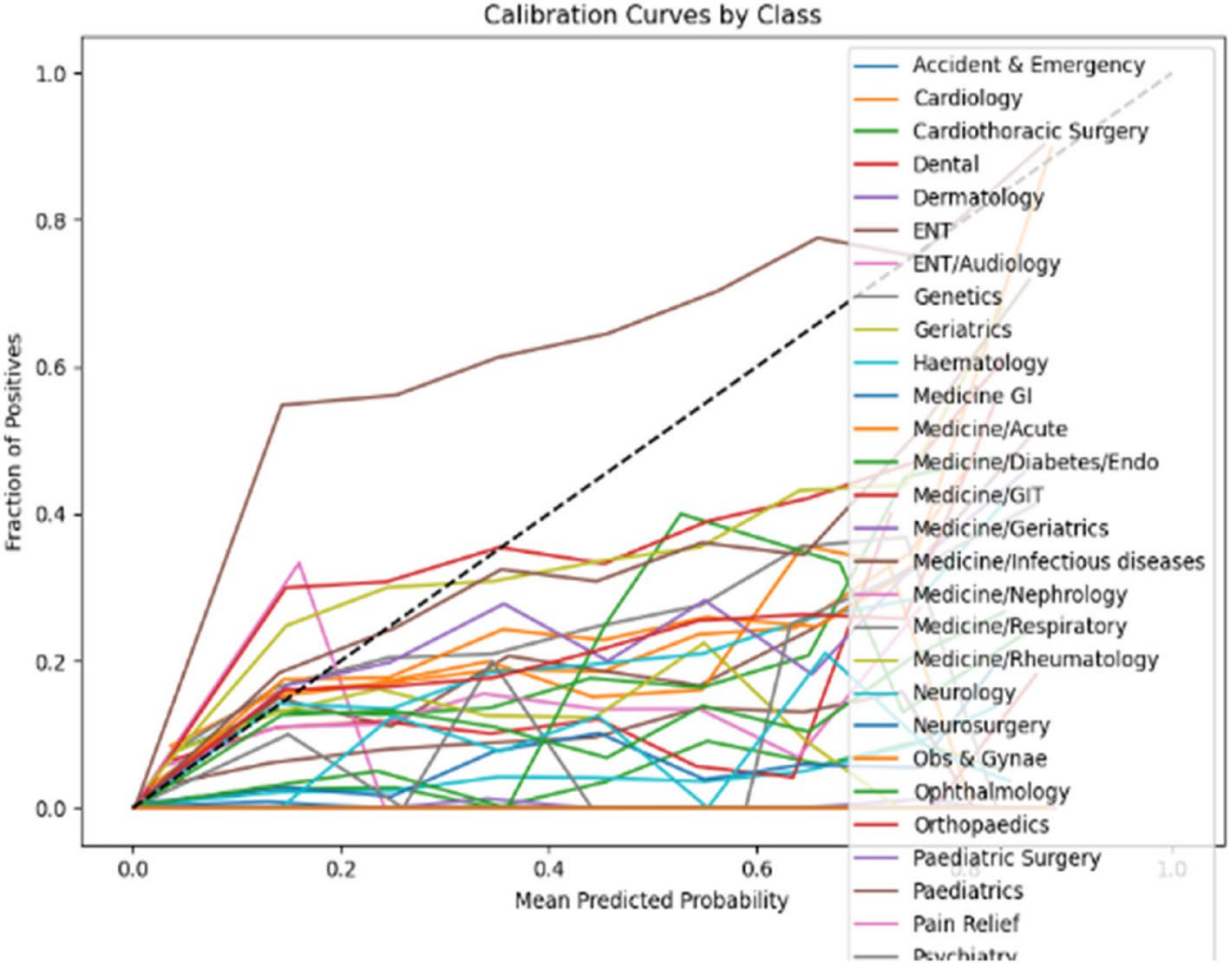
Calibration curve for sub-ward (Stage 1)

**Fig. 19:**
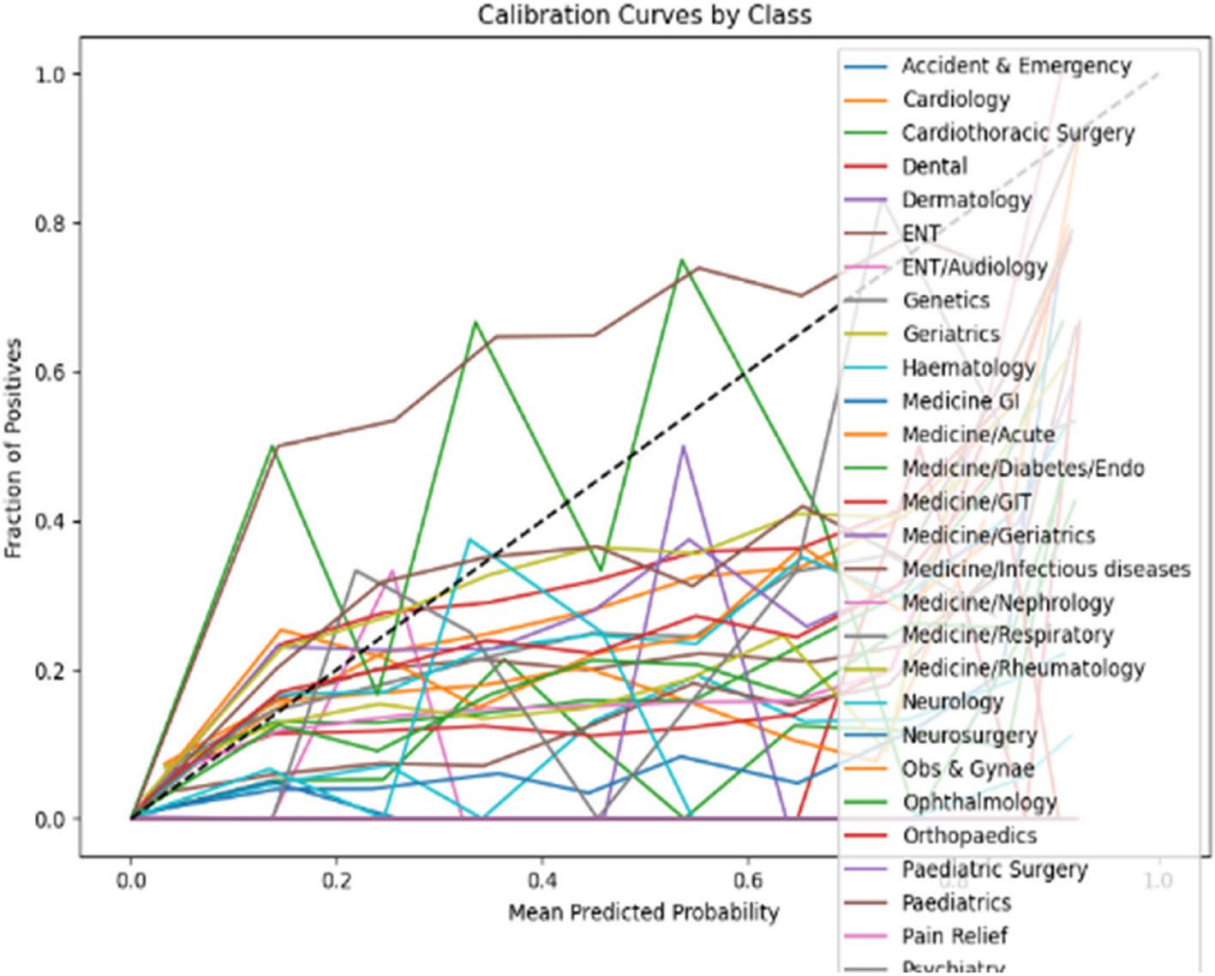
Calibration curve for sub-ward (Stage 2)

The calibration curve analyses for two distinct stages of predictive modelling outlined in Figs. 18 and 19 reveal a notable variation in model performance across various medical specialties. In Stage 1, the calibration curves display significant deviations from the ideal of perfect calibration, particularly for specialties such as Medicine/GI and Neurology, indicating a general underprediction of probabilities and suggesting a lack of adequate data or features within the model. Transitioning to Stage 2, there is a visible improvement in calibration accuracy, especially for fields like Accident & Emergency and Cardiology, which align closer to the perfect calibration line. However, inconsistencies persist with some specialties, such as ENT/Audiology and Dermatology, experiencing dramatic over- or under-estimations at certain probability points. This improvement from Stage 1 to Stage 2 suggests that the integration of additional data or refined modelling techniques has enhanced prediction accuracy, yet the remaining variability across specialties highlights the ongoing need for model tuning and possibly the development of specialty-specific approaches to optimize predictive performance comprehensively.

In both Stage 1 and Stage 2, the most important features for model performance are Main_Complaint_Category and Sub_Category, though their importance slightly decreases in Stage 2. Demographic features such as Age_Group, Gender, and Region maintain relatively stable importance across both stages. However, Stage 2 shows a significant increase in the importance of clinical lab results like Troponin_T_Result, Estimated_GFR_Serum_Result, and C_Reactive_Protein_Result, which were not contributing factors in Stage 1. This shift in Stage 2 highlights the model's increasing reliance on clinical data, improving its predictive capability as these features gain prominence. Meanwhile, features such as Imaging done at ED and Entry_Method see slight increases, reflecting a more nuanced integration of both clinical and administrative data.

### Summary of results

In this study, the overall accuracy of the XGBoost prediction model was chosen as the primary metric to evaluate its performance. Accuracy offers a clear and widely understood measure of the model’s effectiveness in making correct predictions across various tasks, making it suitable for summarising results in a straightforward manner. While additional metrics such as precision or recall could provide deeper insights, overall accuracy is ideal for conveying the general performance of the model in this context.

**Fig. 20:**
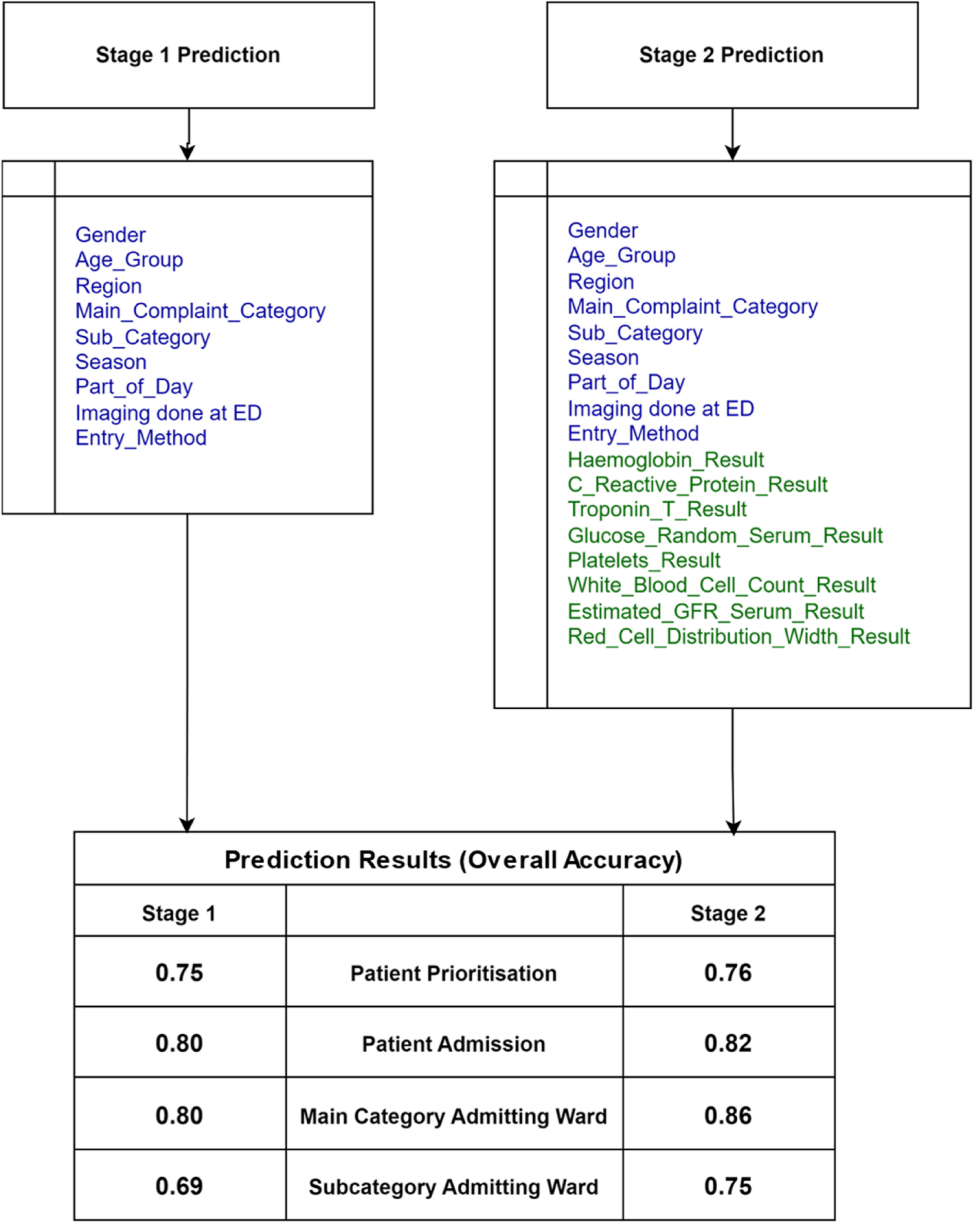
Summary of Prediction Results

The model demonstrated strong predictive capabilities across several key tasks. As outlined in Fig. 20, for patient prioritisation, the model achieved an accuracy of 0.75 in Stage 1, which improved to 0.76 in Stage 2, indicating that the model became more effective at prioritising patients as it evolved. Similarly, in patient admission prediction, the accuracy increased from 0.80 in Stage 1 to 0.82 in Stage 2, reflecting a notable improvement in identifying which patients required admission. When predicting the main category of the admitting ward, the model’s accuracy rose from 0.80 in Stage 1 to 0.86 in Stage 2, showing enhanced reliability in matching patients to the appropriate ward. For the more detailed task of predicting the subcategory of the admitting ward, the model’s accuracy improved from 0.69 in Stage 1 to 0.75 in Stage 2, demonstrating progress in this more complex classification.

These results highlight the model’s ability to improve its predictive performance with further refinement, especially in critical areas such as patient admission and ward categorisation.

## Discussion

This study demonstrates the potential of using machine learning, specifically the XGBoost Classifier, to make accurate predictions in the highly uncertain and dynamic environment of the ED. By successfully predicting patient prioritisation, admission likelihood, and the appropriate admitting ward, the model addresses some of the most pressing challenges in emergency medicine. These predictive capabilities represent a significant advancement, offering a strategic approach to managing ED operations where crowding, human error, and resource limitations are common.

Accurate prioritisation is crucial in emergency settings, where delays in treatment can have serious consequences [51]. Traditionally, patient prioritisation relies heavily on the experience and judgment of triage nurses, which, although effective, is subject to variability and potential bias, especially under stressful and crowded conditions. By accurately identifying patients who need immediate care, this model enables a more efficient triage process, ensuring that critical patients receive the necessary attention without delay. This, in turn, helps reduce the chances of adverse outcomes due to diagnostic mistakes, improving overall patient safety and outcomes.

In addition, predicting patient admission early in their ED visit significantly enhances the effective management of hospital resources. EDs often face challenges in bed availability, leading to extended waiting times and resulting in patient fatigue and exacerbated medical conditions, all of which contribute to negative health outcomes. This model provides hospital administrators and ED staff with the foresight to anticipate admissions and prepare bed availability accordingly. By identifying patients who are likely to require admission, hospital staff can proactively allocate beds, arrange for necessary equipment, and coordinate with inpatient units, thereby reducing bottlenecks in the patient flow. This proactive bed management not only optimises the use of limited hospital resources but also minimises the time patients spend in the ED awaiting admission, reducing overcrowding and the risk of medical errors caused by delayed care. By accurately predicting the correct ward for patient admission, this approach prevents the inconvenience and costs associated with admitting patients to the wrong wards, which often necessitates subsequent transfers. Such transfers increase operational inefficiencies, disrupt care continuity, and further strain hospital resources. Ensuring that patients are placed in the correct ward from the outset enhances both patient outcomes and hospital efficiency.

Additionally, early predictions of the admitting ward offer significant operational advantages. Knowing the likely admitting ward for each patient streamlines care transitions and ensures that the receiving unit is adequately prepared. Bed management nurses can organise the necessary equipment, medications, and specialist staff in advance, promoting continuity of care. This capability also helps avoid costly and disruptive reallocations of patients to different wards, further improving hospital workflow efficiency.

These predictive capabilities are transformative for emergency medicine. By predicting patient prioritisation, admission likelihood, and admitting ward early in the ED process, hospitals can significantly improve patient flow, reduce wait times, and optimise resource allocation. This not only benefits patient care but also addresses systemic issues such as ED overcrowding, which is linked to increased mortality rates and lower quality of care. The ability to anticipate patient needs allows for better staff planning, improved bed utilisation, and more effective communication between ED and inpatient units, all of which contribute to a more efficient healthcare delivery system.

Moreover, the integration of such predictive models into the early stages of emergency care provides healthcare professionals with valuable insights, supporting their decision-making in an environment where rapid and accurate judgments are critical. This model can serve as a supplement to clinical expertise, offering an objective, data-driven approach that reduces the variability inherent in human judgment. While no predictive model can replace the understanding of a trained clinician, the use of machine learning models like XGBoost adds a layer of support that can lead to better informed decisions and more consistent and equitable patient care.

The implications of this study extend beyond individual EDs to the broader healthcare system. By streamlining ED operations and improving patient flow, hospitals can reduce the costs associated with prolonged ED stays, resource wastage due to over-triaging, and delayed treatments. In addition, by minimising crowding and enhancing prioritisation, hospitals can mitigate the risk of diagnostic errors and improve patient outcomes. Furthermore, early prediction allows for more strategic resource management during peak periods, such as flu season or during public health emergencies, enabling a more resilient healthcare response.

In summary, the integration of machine learning models like XGBoost into emergency care processes has the potential to be a game-changer for EDs. By providing accurate, early predictions for patient prioritisation, admission, and admitting ward, this study’s model addresses critical challenges faced by EDs, improving patient outcomes and streamlining hospital operations. The use of such predictive analytics offers a pathway to smarter, more efficient healthcare, where data-driven insights support rapid, informed decision-making. As healthcare systems continue to face growing demands and resource constraints, adopting predictive models could be a key strategy in enhancing the delivery of emergency care.

### Limitations

Despite the significant findings and contributions of this study, several limitations should be acknowledged. Although the dataset spans a five-year period and includes numerous patient visits, it is reliant on data extracted from HIS. HIS data may contain inaccuracies, missing values, or inconsistencies due to human error in data entry, incomplete medical records, or variations in clinical documentation practices. While missing data were addressed through imputation, the approach of filling missing values with zeros may introduce biases or obscure meaningful patterns in the data.

The study utilises a set of 20 features derived from demographic, clinical, and laboratory data. While this is comprehensive, there may be additional factors influencing ED outcomes, such as social determinants of health, environmental factors, or patient-reported symptoms, which were not available or included in the dataset. The exclusion of these variables could limit the predictive power and generalisability of the models.

Although SMOTE was applied to address class imbalance, synthetic oversampling methods have their own limitations [52]. SMOTE generates new data points by interpolating between existing minority class samples, but this may not capture the true complexity of rare events or the underlying distribution of certain target classes. This could lead to overfitting or reduced model performance on underrepresented classes when applied to real-world, unseen data.

XGBoost while highly effective for predictive tasks is inherently a very complex model. Although feature importance can be derived, the decision-making process in this model is not as easily interpretable as simpler models (e.g., logistic regression). This could pose challenges in clinical settings where interpretability and transparency of predictions are crucial for trust and decision-making by healthcare professionals.

The dataset is specific to the Emergency Department of Mater Dei Hospital, a single hospital in one country. Healthcare practices, patient demographics, and hospital policies may differ across regions or countries, limiting the generalisability of the findings to other healthcare settings. Validation of the models using data from different hospitals or healthcare systems would be necessary to confirm their broader applicability.

The dataset covers a six-year period (2017–2022), during which changes in healthcare practices, medical technologies, or hospital policies may have occurred. These changes could affect patient outcomes and the performance of the predictive models. Additionally, the impact of external factors such as the COVID-19 pandemic, which likely influenced healthcare delivery during part of this period, was not explicitly accounted for in the models.

Despite the use of cross-validation and hyperparameter tuning, there is still a risk of overfitting, particularly given the complexity of the models and the large number of features. Overfitting occurs when a model performs well on the training data but poorly on unseen data, limiting its real-world applicability.

Although the data were pseudo-anonymised to preserve patient confidentiality, the use of personal health data for predictive modelling raises ethical concerns. Ensuring that models developed from such data are used responsibly and in ways that benefit patients without compromising privacy is essential.

## Conclusion

This study demonstrates the effectiveness of machine learning models, specifically XGBoost, in predicting key outcomes in the ED. Leveraging a large, comprehensive dataset, the models provide valuable insights into patient prioritisation, hospital admission, and ward allocation. Despite some limitations related to data quality, generalisability, and model interpretability, the findings have the potential to enhance ED efficiency, reduce costs, and improve patient care through better decision-making and resource allocation. In future research, the model can be further enhanced by incorporating additional data fields to improve its predictive accuracy. Including patients past medication history and initial diagnostics taken upon arrival at the emergency department, such as blood pressure, pulse oximetry and Electrocardiography (ECG) results, could provide valuable context for the model. These variables are critical indicators of a patient’s health status and can significantly impact the decision-making process for hospital admissions. By integrating these data points, the model will have access to a more comprehensive view of each patient’s condition when presenting at the ED, potentially leading to more accurate and detailed predictions.

Another area for future exploration involves the acceptance and trust of medical staff towards such predictive models. Will doctors and hospital staff trust and rely on these systems for decision-making? A valuable study would involve surveying medical staff to gauge their confidence in the system, which, although not published in this paper, is planned as part of the PhD study and broader validation exercise. Understanding the perception of the end-users is key to the success of these models in practice.

Additionally, methods for increasing explainability and calculating model uncertainty may further contribute to building trust in the system. While the current prediction model, as outlined in the methods, includes some elements of explainability and aims to reduce the “black box” nature often associated with machine learning models, further work in this area is necessary. Implementing more advanced methods of explainability and uncertainty estimation could provide more transparency in the predictions, offering clinicians better insights into how and why certain decisions are made. Such efforts would not only improve the model’s robustness but also address concerns related to the ethical and responsible use of prediction models in clinical settings.

## Supplementary Information

Supplementary Material 1.

## Data Availability

Numerical data has been intentionally converted to descriptive text to safeguard the sensitive information pertaining to hospital operations. This measure ensures the confidentiality of specific details while still providing a comprehensive overview of the trends and distributions within the data.

## Footnotes

1 https://pandas.pydata.org/
