# Supplementary material for: Predicting Emergency Severity Index (ESI) level, hospital admission, and admitting ward in an emergency department using data-driven machine learning
Source: BMC Med Inform Decis Mak. 2025 Jul 28;25:281. doi: 10.1186/s12911-025-02941-9 (PMC12306103; doi:10.1186/s12911-025-02941-9)
Supplement: Supplementary file 1 — Supplementary Material 1. [file 12911_2025_2941_MOESM1_ESM.docx]

**Appendices**

**Appendix 1 - Descriptive Statistics of the Dataset used in this Study**

**Disclaimer: Numerical data has been intentionally converted to descriptive text to safeguard the sensitive information pertaining to hospital operations. This measure ensures the confidentiality of specific details while still providing a comprehensive overview of the trends and distributions within the data.**

**Gender**

Description: Patient gender

Total records for Gender: 653,127

The dataset indicates a slightly higher proportion of male patients, comprising approximately 52% of the total, compared to female patients who represent about 48%. This suggests a relatively balanced gender distribution within the patient population.

**Age Group**

Description: Patient age group (categorised into 10 groups, each representing a 10-year age range, from 1 to 100 years)

Total records for Age Group: 653,120

Patients aged 71-80 years represent the largest age group seen at the Emergency Department, followed closely by those aged 31-40 years. Individuals aged 61-70 and 41-50 years also constitute a significant portion of visits. There are notable numbers of patients in the 81-90 and 1-10 year age ranges. Patients aged 51-60 and 21-30 years are similarly common, while those in the 11-20 year age group are less frequent. The smallest group consists of those aged 90 years and older.

**Region**

Description: Patient’s registered address (Malta is divided into 5 regions as per National Statistics Office Malta - Regional and Geospatial Statistics)

Total records for Region: 652,437

The data indicates a majority of the patient population is concentrated in the Northern Harbour area, with significant portions also residing in the Southern Harbour and Northern regions. The South Eastern and Western areas have a moderate presence of patients, while Gozo and Comino have a minimal representation.

**Main Category Complaint**

Description: Patient’s complaint recorded during registration (categorised into 13 distinct categories)

Total records for Main Complaint Category: 652,594

This category encompasses the main types of complaints registered at the Emergency Department. The most common complaints include Injuries and Trauma, Gastrointestinal and Abdominal issues, and Specialized Medical Conditions. Other notable categories of complaints involve Cardiac and Circulatory problems, Respiratory and Breathing issues, and Paediatric concerns. The department also handles a considerable number of Medical Investigations, Neurological issues, Obstetrics and Gynaecological complaints, and Urological concerns. Additional, less frequent complaints include Emergency Medical Conditions, Dermatological and Infectious issues, and Abscesses or Lumps.

**Subcategory Complaint**

Description: Patient’s complaint recorded during registration (categorised into 20 distinct categories)

Total records for Subcategory: 652,594

This category details various subcategories of complaints registered at the Emergency Department. Prominent categories include Cardiology, Orthopaedics, and various specialized medical sectors under Medicine such as Acute, Diabetes/Endocrinology, Geriatrics, Gastrointestinal, Infectious Diseases, Nephrology, Respiratory, and Rheumatology. Other significant areas of patient complaints involve Neurology, Paediatrics, and Urology. Specialized surgical complaints are recorded under general surgery, cardiothoracic surgery, neurosurgery, and paediatric surgery, with further specific entries under vascular and plastic surgery. Additional specific complaints categories handled include ENT (Ear, Nose, and Throat), Psychiatry, Obstetrics and Gynaecology, and Ophthalmology. Less common complaints involve areas such as Dermatology, Dental, Pain Relief, and even rarer genetic and thalassaemia-related concerns.

**Entry Method**

Description: Method of arrival at the emergency department.

Total records for Entry Method: 653.127

The vast majority of arrivals were by walking, with a significant number arriving by ambulance. Other modes of arrival, including helicopter, were used much less frequently.

**Admit Status**

Description: Indicates whether patient was admitted to the hospital or discharged from the emergency department (0 – Not Admitted, 1 – Admitted)

Total records for Admit Status: 653,127

The majority of the cases were not admitted, while a substantial portion resulted in admissions.

**Year**

Description: Date and Time (Year) patient visited the emergency department.

Total records for Year: 653,127

The records indicate a gradual increase in occurrences from 2020 through to 2022, with the highest frequency noted in 2017. The year 2019 also saw a significant number of events, closely followed by 2018. The years 2021 and 2020 had comparatively fewer occurrences, with 2020 recording the least.

**Season**

Description: Date and Time (Season) patient visited the emergency department.

Total records for Season: 653,127

The distribution of occurrences across seasons is fairly even, with a slight predominance in the summer, accounting for just over a quarter of the total. Winter and autumn are closely matched, each contributing roughly 25% to the total, while spring sees a slightly lower share, comprising nearly 24%.

**Reg Time**

Description: Date and Time (Time) patient visited the emergency department.

Total records for Reg Time: 653,127

Patient visits peak during mid-morning hours, with the highest number of visits occurring at 10 AM, representing approximately 7.47% of daily visits. This trend continues with high visit rates through the late morning and early afternoon hours. Visits gradually decline in the evening, with a noticeable drop after 8 PM. The lowest frequency of visits is recorded between midnight and early morning, particularly around 4 AM.

**Part of Day**

Description: Date and Time (Part of Day) patient visited the emergency department.

Total records for Part of Day: 65,3127

The majority of events occur in the morning, constituting just under 40% of all occurrences. Afternoon times also see a significant amount of activity, making up approximately 26% of events. Evenings account for just over 20%, while the night period has the fewest occurrences, with roughly 14% of the total.

**ESI Category**

Description: Prioritisation of the patient given by the triage nurse on the urgency of their condition. The data for this category has been categorized into HIGH (ESI 1, 2) and LOW (ESI 3, 4, 5).

Total records for ESI Category: 653,127

The data shows a higher occurrence of instances classified as 'LOW' compared to those categorized as 'HIGH'.

**Admit_main**

Description: Admitting wards patient was admitted to (Admitting wards were classified into main wards and sub-category wards)

Total records for ESI Category: 185,353

Most admissions were to the Medicine ward, representing the majority of cases. Surgery also accounted for a significant portion, followed by Specialty Care. Cardiology, Paediatrics, and Obs & Gynae saw fewer admissions, with Obs & Gynae having the fewest.

**Admit_speciality**

Description: Admitting wards patient was admitted to (Admitting wards were classified into main wards and sub-category wards)

Total records for ESI Category: 185,353

The hospital sees a diverse range of admissions across various specialized wards. Major wards like Medicine/Acute, Medicine/Respiratory, and Medicine/Rheumatology handle a significant portion of admissions, reflecting the hospital's strong focus on comprehensive medical care. Cardiology and Orthopaedics also see a substantial number of patients, underscoring the hospital's capabilities in these specialized areas. Other important areas include Neurology, Paediatrics, and Urology, each addressing specific patient needs. Less common but specialized admissions occur in departments such as Neurosurgery, Psychiatry, and various surgical specialties, including General Surgery and Cardiothoracic Surgery. The smallest number of admissions are noted in highly specialized or specific areas such as Genetics, Thalassaemia, and Pain Relief.

**Appendix 2 - Main and Sub-Category Ward distribution**

| **Main Category Admitting Ward** | **Subcategory Admitting Ward** |
| --- | --- |
| Cardiology | Cardiology |
|  | Cardiothoracic Surgery |
| Medicine | Haematology |
|  | Medicine GI |
|  | Medicine/Acute |
|  | Medicine/Diabetes/Endo |
|  | Medicine/Geriatrics |
|  | Medicine/GIT |
|  | Medicine/Infectious diseases |
|  | Medicine/Nephrology |
|  | Medicine/Respiratory |
|  | Medicine/Rheumatology |
|  | Neurology |
|  | Psychiatry |
|  | Thalassaemia |
| Obs & Gynae | Obs & Gynae |
| Paediatrics | Paediatric Surgery |
|  | Paediatrics |
| Specialty Care | Dental |
|  | Dermatology |
|  | ENT |
|  | ENT/Audiology |
|  | Genetics |
|  | Geriatrics |
|  | Neurosurgery |
|  | Ophthalmology |
|  | Orthopaedics |
| Surgery | Accident & Emergency |
|  | Pain Relief |
|  | Surgery general |
|  | Surgery plastics |
|  | Surgery/acute |
|  | Surgery/NSU |
|  | Surgery/Plastics |
|  | Surgery/Vascular |
|  | Urology |
